# Identification of CCp5 and FNPA as Novel Non-canonical Members of the CCp Protein Family in *Babesia bovis*

**DOI:** 10.3389/fvets.2022.833183

**Published:** 2022-02-15

**Authors:** Sezayi Ozubek, Heba F. Alzan, Reginaldo G. Bastos, Jacob M. Laughery, Carlos E. Suarez

**Affiliations:** ^1^Department of Veterinary Microbiology and Pathology, College of Veterinary Medicine, Washington State University, Pullman, WA, United States; ^2^Department of Parasitology, Faculty of Veterinary Medicine, University of Firat, Elâzig, Turkey; ^3^Parasitology and Animal Diseases Department, Veterinary Research Institute, National Research Center, Giza, Egypt; ^4^Animal Disease Research Unit, Agricultural Research Service, United States Department of Agriculture, Pullman, WA, United States

**Keywords:** *Babesia bovis*, bovine babesiosis, sexual stages, transmission blocking vaccine, CCp protein family

## Abstract

Bovine babesiosis, caused by *Babesia bovis*, is an economically significant tick-borne disease that imposes restrictions to livestock production worldwide. Current methods to control bovine babesiosis have severe limitations and novel approaches, including transmission-blocking vaccines, are needed. Members of the widely conserved CCp family are multidomain adhesion proteins containing LCCL motifs, which are differentially expressed on gametocytes of apicomplexans, including *Babesia* spp. and *Plasmodium* spp. While *Plasmodium* parasites contain 6 distinct *CCp* genes, only three members (CCp 1-3) were previously identified in *B. bovis*. In this study, we describe the identification and characterization of two novel non-canonical members of the *CCp* gene family in *B. bovis*, named CCp5 and FNPA. The genes were identified *in silico* by TBLASTN using *P. falciparum* CCp family domains as queries. Unlike CCp1-3, the *B. bovis* CCp5 and FNPA proteins lack the LCCL canonical domain but contain other typical multidomain adhesion motifs which are present in classical CCp proteins. In addition, the *B. bovis CCp5* and *FNPA* are in synteny with known *CCp* genes in related apicomplexans. Sequence analysis of these two proteins demonstrated high sequence conservation among *B. bovis* different isolates. Transcription, immunoblot, and immunofluorescence analyses demonstrated expression of CCp5 and FNPA in blood and *in vitro* induced sexual stages of *B. bovis*. The FNPA, in contrast to CCp5, has a predicted transmembrane domain, suggesting that it might be expressed in the surface of sexual stage parasites. Altogether, finding of this study support FNPA as a possible target of a transmission-blocking vaccine against *B. bovis*.

## Introduction

*Babesia bovis*, which is transmitted by *Rhipicephalus* spp. ticks, is a hemoparasite responsible for bovine babesiosis, a disease that causes enormous economic losses to the cattle industry in tropical and subtropical regions worldwide. *Babesia* parasites have a complex lifecycle that includes the development of asexual stages in vertebrate hosts and sexual stages inside their definitive tick vectors (1–3). Sporozoites, the infectious form of *B. bovis*, are introduced via tick saliva into the bovine host by infected *Rhipicephalus* spp., where they invade and reproduce asexually as merozoites in red blood cells (RBC). Upon feeding on infected cattle, ticks may ingest *B. bovis*-infected RBCs and *B. bovis* gametogenesis is induced while in the tick midgut lumen, leading to zygote formation (4, 5). In some species of *Babesia*, gametocytes have been identified in host RBCs, but it is impossible to distinguish them under the light microscope (6, 7). *Babesia bovis* acute infection in cattle results in high fever, anorexia, inappetence, and severe intravascular hemolytic anemia. In addition, *B. bovis* expresses proteins that facilitate cytoadhesion of infected RBCs to capillaries, causing neurological symptoms and general organ failure, leading to rapid death of cattle, especially in immunologically naïve adult (more than one-year old) animals (3, 8, 9).

Commonly used methods to control acute bovine babesiosis combine approaches for tick management, immunization with live, attenuated, blood based *Babesia* vaccines, and babesicidal drugs. Despite being relatively effective, these interventions have various disadvantages and could be improved. Production of live *B. bovis* vaccines is expensive and laborious, requiring serial rapid passages of virulent strains in splenectomized bovines to obtain an attenuated parasite strain. In addition, these vaccines require a cold chain for deployment, are often transmissible by competent ticks, and there is a risk for the parasites to revert to virulence (1–3, 8). Thus, alternative subunit vaccines that can contribute toward effective control and eradication of bovine babesiosis are urgently needed. The current arsenal of measures against bovine babesiosis could also be greatly enhanced by the addition of transmission-blocking vaccines (TBV). Such TBV could be based on parasite sexual stage proteins, such as HAP2 (10), members of the CCp family (11) and 6cys (12–14).

CCp is highly conserved family of six multidomain adhesion proteins, containing LCCL motifs, which are differentially expressed on the surface of apicomplexan gametocytes. Previous experiments performed in the *Babesia*-related *Plasmodium* parasites demonstrated that knocking out *CCp* genes leads to the blocking of sexual stage development of the parasite in the mosquito vector (15–17). Three *CCp* genes, denominated *CCp1-3*, were previously identified in *B. bovis* and *B. bigemina* (11). In the current study we describe the identification of two novel genes of the *B. bovis* CCp family, which we named *CCp5* and *FNPA*. The newly identified *B. bovis* FNPA and CCp5 proteins lack the LCCL domain characteristic of the other members of the CCp family, but do contain other functionally relevant motifs found in *Plasmodium* CCps (PfCCps), and their coding genes have conserved synteny with other *CCp* genes in related apicomplexans. Therefore, we propose CCp5 and FNPA as non-canonical members of the *B. bovis* CCp family. The findings of this work contribute toward a better understanding of the biology of *B. bovis* and support the rationale for using members of the CCp family in the development of novel TBV against *B. bovis*.

## Materials and Methods

### *In silico* Genes Identification by Genomic Search and Bioinformatics Analysis

The novel members of the *CCp* gene family were identified by performing BLAST searches (http://blast.ncbi.nlm.nih.gov/Blast.cgi) of the *B. bovis* genome using the amino acid sequences of CCp5/LAP3 (GenBank ID XP_001351021.1), and FNPA/LAP5 (GenBank ID XP_001348665.2) of *P. falciparum* 3D7 strain (18) as queries. Multiple alignments of amino acid sequences were generated using Clustal Omega Multiple Alignment (http://www.ebi.ac.uk/Tools/msa/clustalo). Phylogenetic tree prediction was generated by MEGAX program (19). Simple Molecular Architecture Research Tool “SMART” program (http://smart.embl-heidelberg.de), PROSITE program (https://prosite.expasy.org) and the Transmembrane Hidden Markov Model package 2 (TMHMM2) (http://www.cbs.dtu.dk/services/TMHMM-2.0) were used to predict domains and signal peptides in the CCp protein sequences. Synteny studies were carried out using the Piroplasma DB database (piroplasmadb.org/piro/app).

### *Babesia bovis in vitro* Culture

The *B. bovis* S79-T3Bo strain was propagated in continuous microaerophilic stationary-phase culture, as previously described (10, 14) and used for gene and protein expression analyses. Genomic DNA from the *B. bovis* strains T2Bo (20), Mo7 (21, 22), and L17 (20) were used for gene amplification by PCR and followed by comparison of *CCp5* and *FNPA* gene sequences.

### Polymorphism and Genetic Analysis

PCR products derived from the full length *CCp5* and *FNPA* genes from different strains of *B. bovis* were cloned into pCR-TOPO 2.1 vector (Invitrogen, CA, USA) and sequenced (Eurofins MWG Operon, Louisville, KY). Sequencing of PCR products was performed using the primers indicated in the Supplementary Table 1. The complete gDNA sequence for the newly identified genes were compared among four geographically distinct *B. bovis* strains including Texas (T2Bo) attenuated, T2Bo virulent (20), Mo7 (21, 22), and L17 (Argentina) (20). Strain-specific single nucleotide polymorphisms (SNPs) were then estimated to calculate the ratio of synonymous to non-synonymous changes (12–14). SNAP (http://hcv.lanl.gov/content/sequence/SNAP/SNAP.html) was used to investigate ω (dN/dS ratio) as follows: ω > 1 indicates positive selection, as the selection has caused some amino acid substitution; and ω <1 indicates occurrence of purifying selection and a high degree of sequence conservation (23). The Multiple Alignment using Fast Fourier Transform (MAFFT) (24) was used for DNA sequence alignment. The Molecular Evolutionary Genetics Analysis (MegaX) (19) was used to generate phylogenetic relationship among CCp family members. Nucleotide substitutions were manually calculated.

### *In vitro* Induction of *B. bovis* Sexual Stages

*Babesia bovis* infected-RBCs were induced to form sexual stages *in vitro* using 100 μM xanthurenic acid (XA) (Sigma, St. Louis, MO, USA), as previously described (10, 25). Cultures were incubated up to 48 h at 27 C with 5% CO_2_, and collected at 0, 12, 24- and 48-h post-induction for RNA extraction. In addition, blood smears were prepared from induced and non-induced parasite cultures at different time points and stained with Giemsa stain to be further analyzed under light microscope for morphological changes.

### RNA Isolation, CDNA Synthesis and Transcription Analysis for *CCp* Gene Family Members

Total RNA was extracted from induced and non-induced sexual stage *B. bovis* cultures. Parasites were collected in Trizol (Thermo Fisher Scientific, Waltham, MA, USA) and RNA extractions were performed using a phenol-based protocol. Two hundred ng of total RNAs were utilized for cDNA synthesis using the Superscript III™ cDNA Synthesis Kit (Thermo Fisher Scientific, Waltham, MA, USA) following the manufacturer's protocol. Synthesized cDNA was used for PCR and quantitative PCR (qPCR) to test the transcript levels of *CCp5* and *FNPA* in *B. bovis* parasite. The qPCRs were performed in a CFX96? Real-Time PCR Detection System using the SsoFast™ EvaGreen^®^ Supermix (Bio-Rad, USA). The cycling conditions consisted of an enzyme activation step of 95°C for 30 s followed by 40 cycles of 95°C denaturation for 5 s and annealing/extension of 60°C for 5 s. Reactions were performed in duplicate in 20 μl using 200 nM of each primer and 2 μl of a 1/20 dilution of cDNA as template. The *B. bovis gapdh* gene (BBOV_II002540) was used as a reference gene and transcription level of *CCp* genes was normalized to blood stage (non-induced) parasites using the formula: relative expression (sample) = 2^[Cq(control)−*Cq*(*sample*)]^, where time zero was used as Cq control. Supplementary Table 1 shows the primers used to evaluate the cDNA levels for *CCp5* and *FNPA*. RT-PCR amplicons were cloned into pCR-TOPO 2.1 vector (Invitrogen, CA, USA) and sequenced (Eurofins MWG Operon, Louisville, KY). *CCp5* and *FNPA* transcript levels were compared in blood and kinete stages using previously reported *B. bovis* RNAseq datasets (26).

### Synthetic Peptide Design and Polyclonal Antibody Generation

Synthetic peptides ranging from 20 to 25 amino acids were produced based on the sequence of CCp5 and FNPA, as follows: Bo-CCp5 SRRHVTNASFSLFEDPSDSSNSDTS (aa16–40); and Bo-FNPA—GDINQDVVSPKDFTIPSGTD (aa 43–62). Peptides conjugated with keyhole limpet hemocyanin (KLH) were used for the immunization of rabbits (*n* = 2) as previously described (10, 11, 14) (Pacific Immunology Ramona, CA, USA) (protocol #11/30/20. Ref. SOP-1 for CCp5, protocol #12/28/20. Ref. SOP-1 for FNPA). The rabbit polyclonal antibodies were used in immunoblot and immunofluorescence assays to investigate the expression of the CCp5 and FNPA proteins.

### Immunoblot Assays

Immunoblots were performed using a standard protocol, as previously described (11). Briefly, membranes were blocked in 5% non-fat milk and incubated separately with anti-CCp5 rabbit polyclonal antibodies (1:50), anti-FNPA rabbit polyclonal antibodies (1:50), and pre-immune polyclonal rabbit serum (1:50). The membranes were incubated with an appropriate HRP conjugated secondary antibody (goat anti-rabbit IgG peroxidase conjugate; Life Biosciences, Boston, MA, USA). The immune complexes were revealed using an enhanced chemiluminescence method (ECL™) (Amersham).

### Immunofluorescence Assays (IFA)

Non-induced and induced *B. bovis* cultures were collected at 0, 12, 24, and 48 h and used for IFA. Cells were washed with 1X PBS at 900xg and pellets suspended with 1X PBS containing 3% bovine serum albumin (BSA). A smear was made by taking 5 μl from each cell preparation to a glass microscope slide. The slides were air dried, fixed for 5 min in cold acetone, and blocked with 1X PBS containing 10% BSA for 30 min. Then, slides were incubated with rabbit anti-CCp5 or -FNPA sera (1:50) in 1X PBS-10% BSA for 1 h. The slides were washed 3 times with 1X PBS and incubated in 10% BSA with goat anti-rabbit IgG AlexaFluor^®^555 (Thermo Fisher, Waltham, MA, USA), and then again washed 3 times with 1X PBS and mounted with a drop of Prolong™ Gold Anti-fade with 4′,6-diamidino-2-phenylindole (DAPI) (Thermo Fisher, Waltham, MA, USA) and cover slip. The slides were analyzed using a Leica Sp8-X White Light Laser point scanning confocal microscope (Leica Microsystems, Wetzlar, Germany).

## Results

### Identification of Two New Non-canonical *CCp* Genes in *B. bovis*

**Figure 1:**
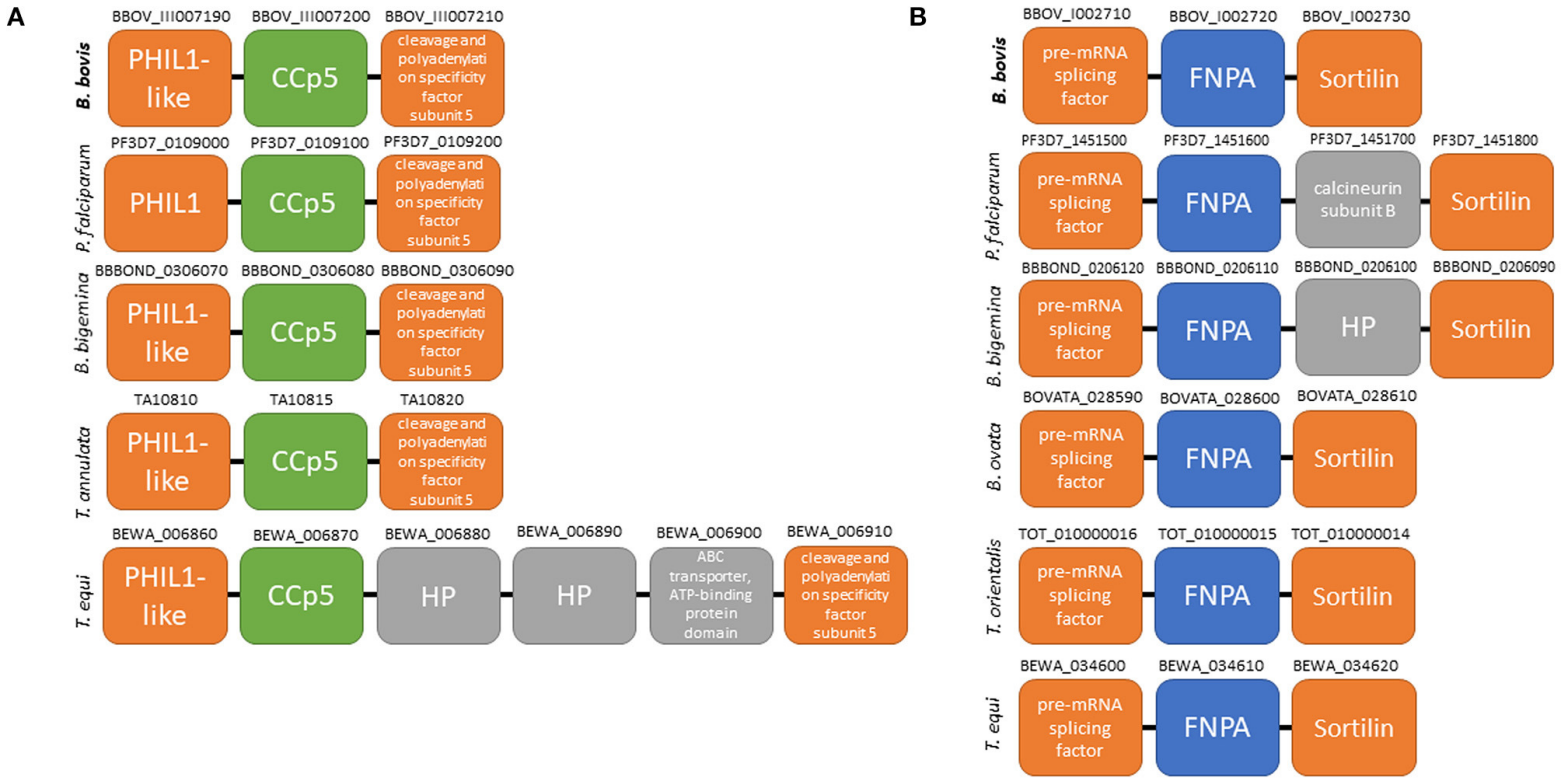
Schematic representation of gene localization and synteny maps of the *B. bovis CCp5* **(A)** and *FNPA* **(B)** genes (bold) among several piroplasm species.

Three *CCp* gene family members were previously identified in *B. bovis* and denominated *CCp1, CCp2* and *CCp3* (11). However, database searches on the *B. bovis* genome using TBLASTN with the *P. falciparum* CCp family domains as queries resulted in the identification of two additional and previously unnoticed *CCp-*like genes, which we termed here as *CCp5* and *FNPA*. The *CCp5* and *FNPA* genes were originally annotated as BBOV_III007200 and BBOV_I002720 (PA14 domain containing proteins), respectively (Table 1). Single-copy *B*. *bovis CCp5* and *FNPA* genes are located between positions 1,564,074 and 1,567,232 bp of chromosome 3, and positions 270,945 and 273,803 bp of chromosome 1, respectively, as schematically shown in Figure 1. While the *B. bovis CCp5* gene lacks introns, the *FNPA* gene contains three introns (Table 2). The *CCp5* and *FNPA* genes encode for proteins with predicted molecular weights of 117.5 and 98.9 KDa, respectively (Table 2).

**Table 1:** Comparison of CCp proteins among *P. falciparum* and *B. bovis* parasites.

**Gene**	**Species**	**Gene ID**	**Number of LCCL domains**	**Signal peptide**	**Number of TM domains**
CCp1	*P. falciparum*	PF14_0723	1	Yes	No
	*B. bovis*	BBOV_III006360	1	Yes	No
CCp2	*P. falciparum*	PF14_0532	1	Yes	No
	*B. bovis*	BBOV_II003700	1	No	No
CCp3	*P. falciparum*	PF14_0067	4	Yes	No
	*B. bovis*	BBOV_III008930	4	No	No
CCp4	*P. falciparum*	PFI_0185w	1	Yes	No
	*B. bovis*	NF	NF	NF	NF
CCp5	*P. falciparum*	PFA0445w	1	Yes	No
	*B. bovis*	BBOV_III007200	0	No	No
FNPA	*P. falciparum*	PF14_0491	0	Yes	No
	*B. bovis*	BBOV_I002720	0	No	Yes

*NF, not found; TM, transmembrane domains*.

**Table 2:** Predicted features of the *B. bovis CCp5* and *FNPA* genes.

**Gene**	**Chromosome**	**Gene length (nt)**	**Transcript size (nt)**	**Number of introns**	**Protein length (aa)**	**Protein KDa**
CCp5	3	3,159	3,159	0	1,052	117.5
FNPA	1	2,859	2,747	3	886	98.9

*Nt, nucleotide; aa, amino acid; KDa, kilodalton*.

Synteny maps for *CCp5* and *FNPA* genes show conservation among several distinct piroplasm species, including *B. bigemina* (Figure 1). However, similar homology and synteny analysis searches failed in identifying a gene homologous to *CCp4* in the *B. bovis* genome (Supplementary Figure 1).

**Figure 2:**
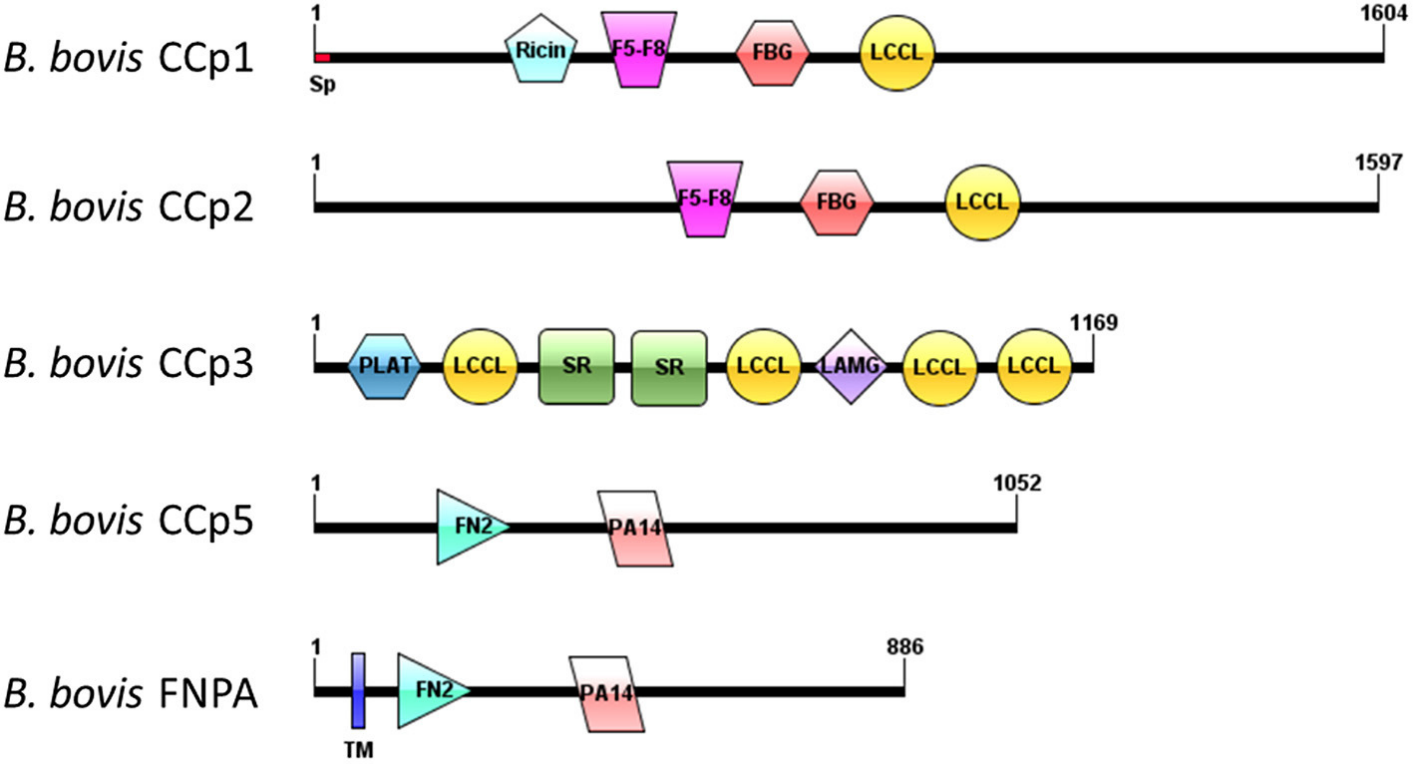
Schematic domain architectures representation of all CCp family members of *B. bovis*, including the novel non-canonical members CCp5 and FNPA. Diagrams were constructed using IBS program (28), version 1.0. Domain abbreviations are as follows: Sp, signal peptide; Ricin, Ricin domain, F5-F8, F5-F8 type C domain, FBG, Fibrinogen-related domains; LCCL, Limulus coagulation factor domain; PLAT, Polycystin-1, Lipoxygenase, Alpha-Toxin; SR, Scavenger receptor domain; LAMG, Laminin G domain; FN2, Fibronectine type 2 domain; PA14, Anthrax PA domain; TM, transmembrane domain.

**Figure 3:**
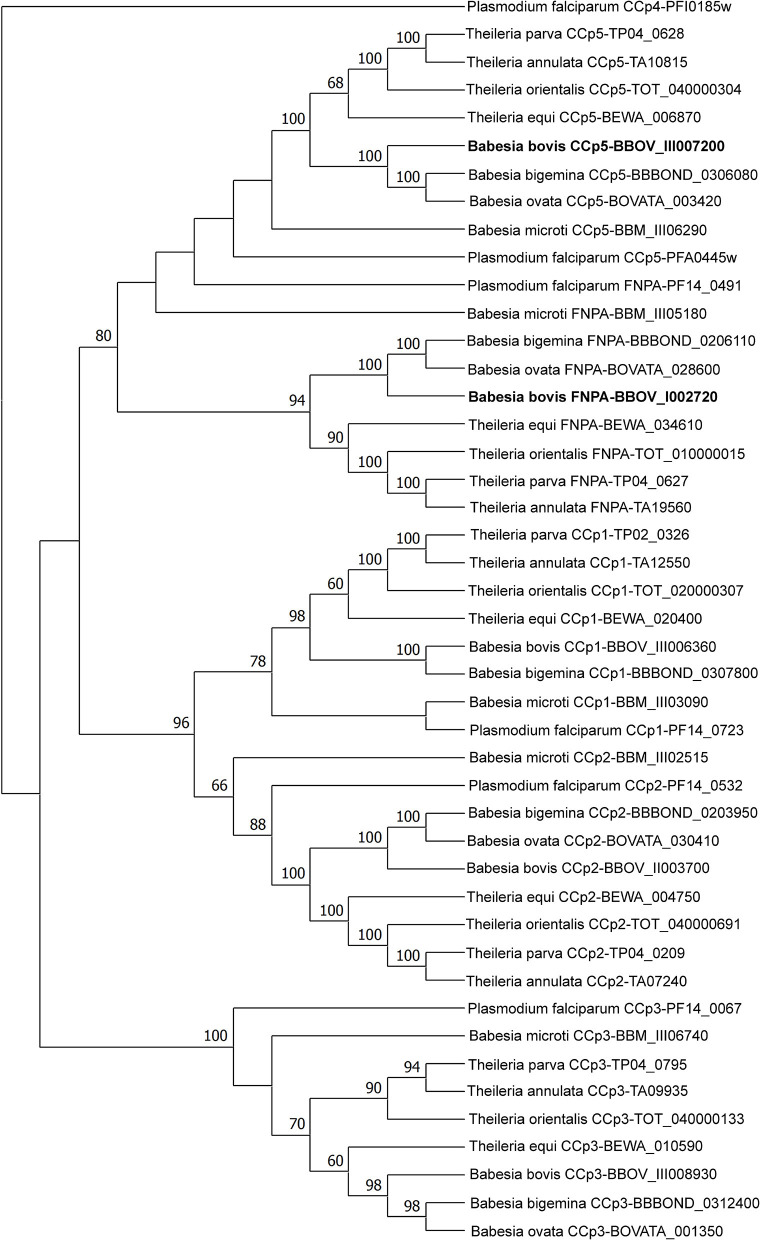
Phylogenetic analyses of piroplasm CCp family protein sequences by maximum likelihood method based on the Whelan and Goldman + Freq. model (29). Sequences were obtained from the GenBank database. The newly identified *B. bovis CCp5* and *FNPA* genes are shown in bold. The *CCp4* gene sequence of *P. falciparum* was used as outgroup.

Analysis of domain architectures, using two different programs, confirmed the lack of LCCL domains in the CCp5 and FNPA proteins in *B. bovis*, However, a fibronectin type 2 domain (FN2), a collagen-binding domain, followed by a domain (PA14), similar to anthrax protective antigen, with potential roles in carbohydrate recognition were found in identical arrangements among these two proteins (Supplementary Figure 2). These domains are known to mediate calcium dependent interactions (27). A transmembrane domain was found only in *B. bovis* FNPA (Table 1; Figure 2), suggesting that this protein might be inserted in the cell membrane, and the possible exposure of functionally relevant protein domains on the surface of the parasite. A phylogenetic analysis performed with the five known CCp members of *B. bovis* together with similar CCp proteins of other related piroplasm species showed formation of five different clades corresponding to CCp1-3, FNPA, and CCp5 (Figure 3). Interestingly, a similar domain architecture search performed on the FNPA and CCp5 proteins of *B. bigemina* and *Theileria* also failed in detecting recognizable LCCL domains. Therefore, database searches, together with phylogenetic and synteny analysis, support the notion presented hereby that *B. bovis* FNPA and CCp5 are true homologs of the FNPA and CCp5 proteins of other related apicomplexan organisms. Also, results suggest that canonical LCCL domains might not be present in the CCp5 and FNPA proteins in *Babesia* and *Theileria* organisms.

### *Cp5* and *FNPA* Genes Are Highly Conserved Among Geographically Distinct *B. bovis* Strains

The complete gDNA sequence for *CCp5* and *FNPA* genes was compared among four different *B. bovis* strains including T2Bo attenuated, T2Bo virulent, Mo7, and L17 (Argentina). Sequence comparisons showed that *CCp5* and *FNPA* genes are highly conserved among these four *B*. *bovis* strains (96 to 100% identity) (Supplementary Figure 3). Moreover, the calculated synonymous and non-synonymous S/N ratios with the parameter, ω (ω = dN/dS), as an indicator of potential selection pressures are shown in Table 3. In all cases, ω parameters <1 was obtained, providing no support for positive selection for the *CCp5* and *FNPA* genes. Altogether, these observations suggest that these two genes are not likely exposed to immune selection in the vertebrate host. The full DNA sequences of the CCp5 and FNPA genes derived from these four distinct strains, and their alignment are shown in Supplementary Figures 4A,B, respectively. In addition, the CCp5 and FNPA genes are also well-conserved among different *Babesia* spp. and other piroplasm species (Supplementary Figure 5).

**Table 3:** Polymorphism and average SNPs of the *CCp5* and *FNPA* genes among different distinct *B. bovis* strains.

	**CCp5**	**FNPA**
Nucleotide substitutions	184	85
Average dN	1.6265	0.8548
Average dS	2.0505	0.9634
ω dN/ dS	0.79	0.89
pn/ps	0.97	0.91

### *CCp5* and *FNPA* Genes Are Expressed in Blood and Sexually Induced Stages of *B. bovis*

**Figure 4:**
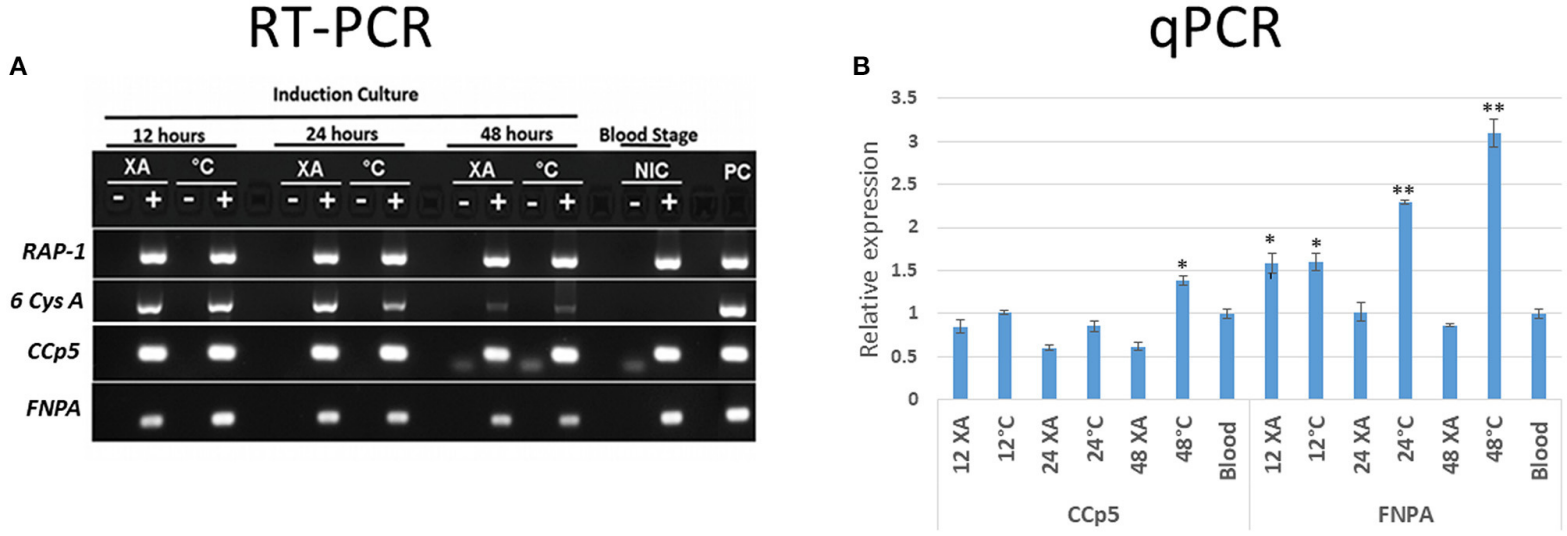
**(A)** The RT-PCR analysis for the detection of *RAP-1, 6-Cys A, CCp5* and *FNPA* transcripts was performed using non-induced (blood stage-NIC) and induced (12, 24 and 48 h) *in vitro* cultures (XA, xanthurenic acid induction; C, temperature induction; PC, positive control. **(B)** Relative expression of *B. bovis CCp5* and *FNPA* by non-induced (blood stage) and induced stages (12, 24, and 48 h) *in vitro* cultures. *p = 0.0.012–0.0028; **p < 0.0001 (*p* value compared with blood stage).

The levels of *CCp5* and *FNPA* transcripts were investigated by RT-PCR analysis performed on RNA extracted from cultured non-induced and *in vitro* sexual-stage induced *B. bovis* parasites. The *RAP-1* gene was also similarly amplified by nested PCR as control for cDNA quality of all stages (30), and the sexual stage marker *6cysA* gene (14) was amplified for sexual stage development control (Figure 4A). The qPCR transcription analysis demonstrated differential expression of *CCp5* and *FNPA* genes in blood and sexual stages (Figure 4B). The transcription levels of *CCp5* were similar between sexually induced and blood stage parasites at 12 and 24 h post-sexual stage induction, but it significantly increased in sexually induced parasites at 48 h post induction. However, the levels of expression of the *FNPA* gene significantly increased at 12, 24 and 48 h post-sexual stage induction, compared to the non-induced blood stage parasites. The peak for *FNPA* gene expression was observed at 48 h after sexual stage induction by temperature.

**Figure 5:**
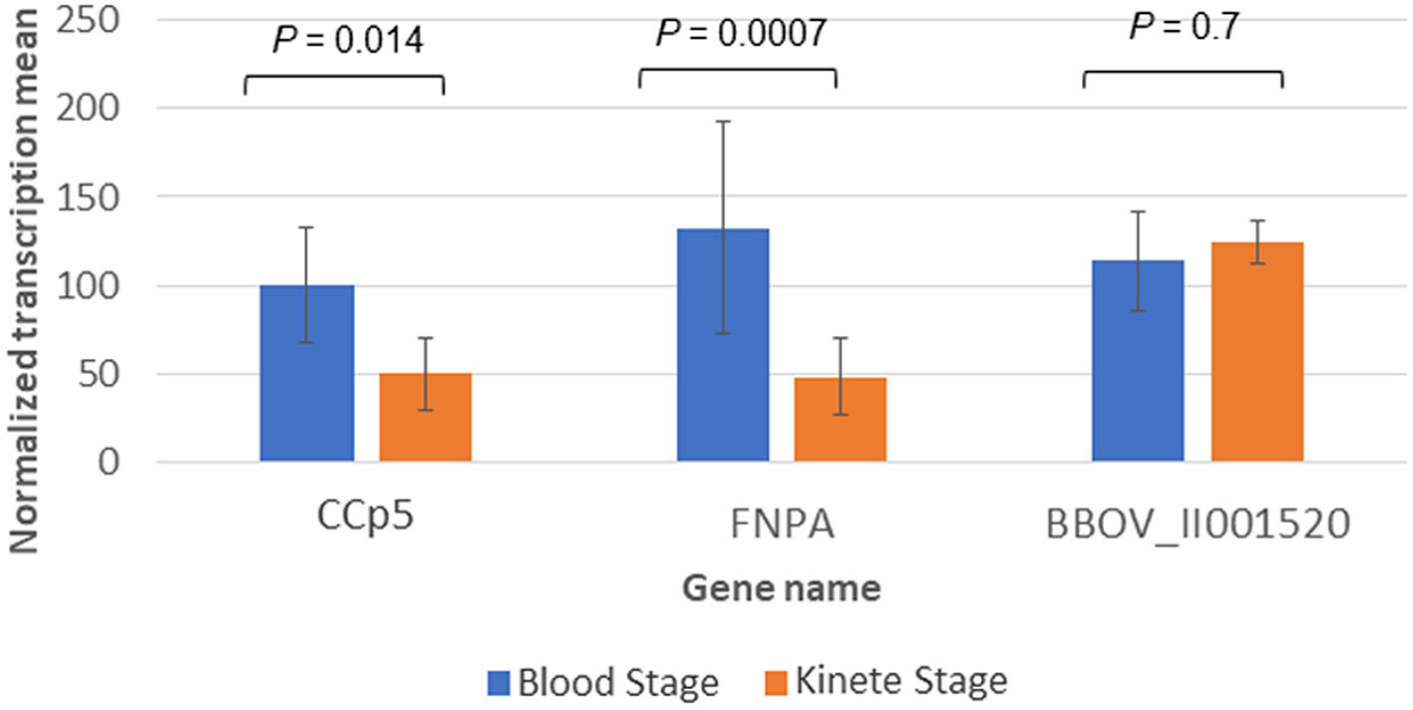
Differential expression of *CCp5* and *FNPA* between *B. bovis* blood and kinete stages, respectively in the RNA-Seq datasets. The *26S proteasome non-ATPase* gene (BBOV_II001520) was used as a control gene for blood and kinete stages.

In addition, the *B. bovis* RNAseq datasets were used to compare the transcript levels of *CCp5* and *FNPA* at the blood and kinete stages (Figure 5). Overall, these comparisons showed that the blood stage levels of *CCp5* and *FNPA* transcript are significantly higher than the kinete stage levels (Figure 5).

**Figure 6:**
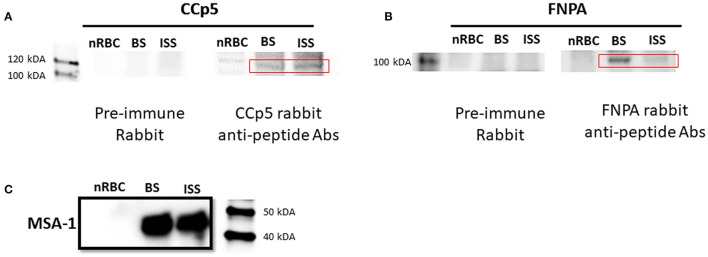
Immunoblot analysis using rabbit polyclonal antibodies against CCp5 peptide **(A)** and FNPA peptide **(B)** performed on blood stage (BS) and induced sexual stage (ISS) *B. bovis* parasites. The monoclonal antibody BABB35, reactive with MSA-1, was used as a control **(C)**.

Expression of CCp5 and FNPA proteins were also examined in *in vitro* non-induced and sexual stage induced (48 h-temperature induction) parasites by immunoblot analysis using rabbit polyclonal anti-CCp5 and FNPA peptides (Figure 6). Results showed that anti-CCp5 and anti-FNPA peptide antibodies weakly reacted with blood stage proteins of expected molecular weight of 117 and 97 KDa, respectively (Figures 6A,B). In contrast, reactivity of the monoclonal antibody BABB35 against MSA-1 was strongly evident in both, blood and sexual stage lysates (Figure 6C). Therefore, consistent with the transcription analysis, CCp5 and FNPA protein expression were also confirmed in blood and sexual stage parasites.

**Figure 7:**
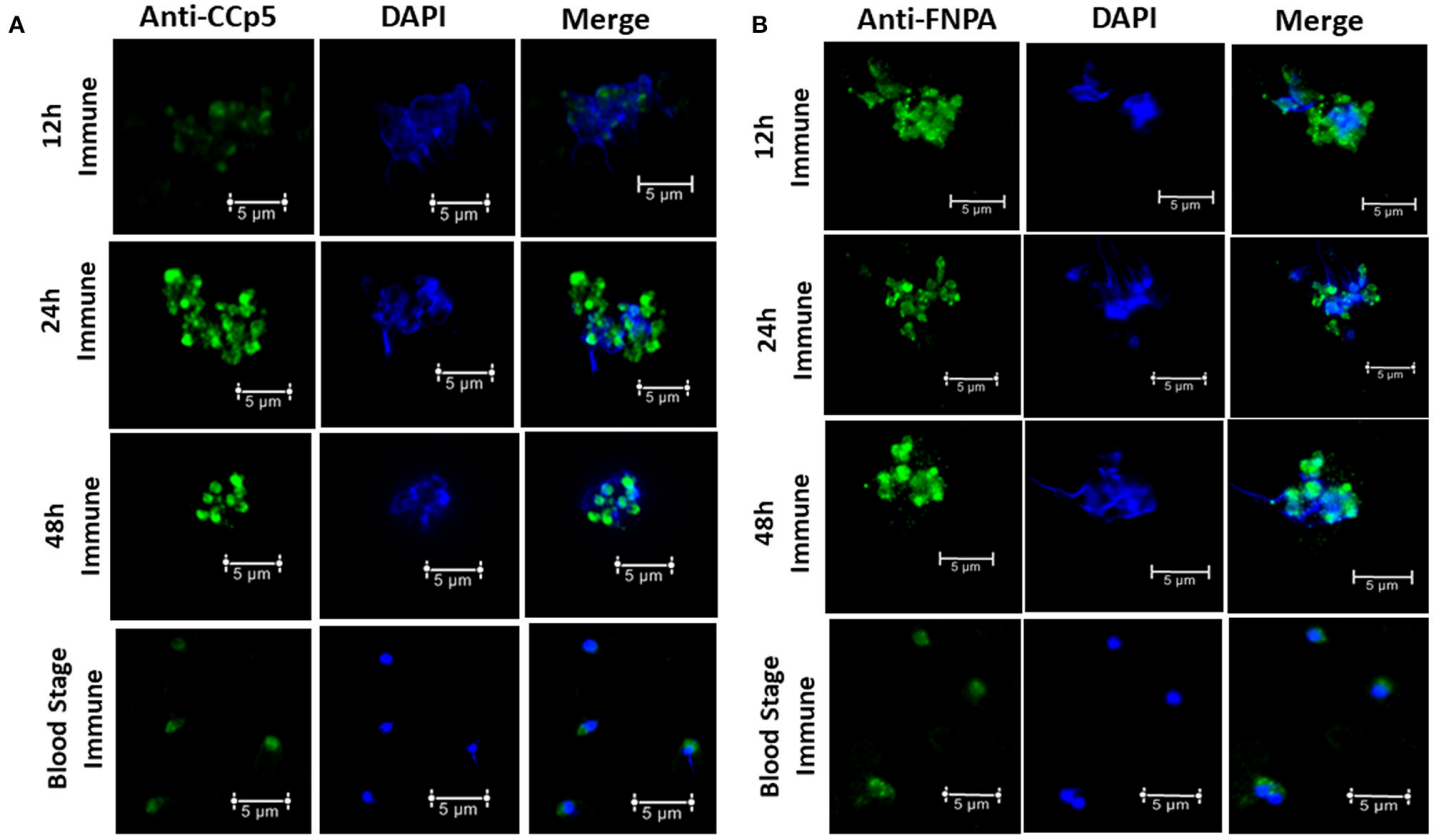
Immunofluorescence assays demonstrating the expression of CCp5 **(A)** and FNPA **(B)** in blood stage and *in vitro* induced sexual stages (12–24–48 h) of *B. bovis*.

Immunofluorescence assays using rabbit polyclonal anti-CCp5 and FNPA peptides also revealed expression of CCp5 and FNPA proteins in *in vitro* non-induced (blood stage-BS) and induced (12–24–48 h-XA induction) parasites. Fluorescence signals were more intense for sexually *in vitro* induced forms of the parasite compared to blood stage or non-induced forms (Figure 7). Pre-immune rabbit sera did not show any IFA reactivity against blood stage or *in vitro* induced sexual stages (Supplementary Figure 6).

## Discussion

The CCp protein family, which has an important role in sporozoite development and infectivity, consists of six members with a modular structure composed of multiple protein, lipid and carbohydrate binding domains, including LCCL domains in *Plasmodium* parasites. However, while the *Pf* CCp1-*Pf* CCp5 proteins contain at least one LCCL domain, *Pf* FNPA, which lacks such domain, has also been included as a member of the *Pf* CCp protein family due to its structural similarity to *Pf* CCp5 (15–17). The FNPA acronym is derived from the domains FN2 and PA14 present in this proteins (15). Although six LCCL protein family members have been identified in *P. falciparum*, only genes encoding homologs of CCp1-3 proteins have been reported in *B. bovis* and *B. bigemina* so far (11). In this study, we describe the presence of two additional single gene-copy *CCp*-related genes (*CCp5* and *FNPA*) in the genome of *B. bovis* and *B. bigemina*, as previously described in *Plasmodium* spp. (16, 31). While *B. bovis* FNPA is structurally similar to *Pf* FNPA, the *B. bovis, B. bigemina and T. equi* CCp5 proteins contain no canonical LCCL domains, unlike *Pf* CCp5. However, the *B. bovis* CCp5 clusters with other *Babesia* sp. and *Plasmodium* parasites in the phylogenetic analysis presented in this study. This finding, together with conserved synteny, supports the denomination of this protein as *B. bovis* CCp5, despite the lack of the canonical LCCL domain.

Interestingly, no genes encoding for a protein equivalent to *Pf* CCp4 were found in the genome of *Babesia* parasites, either by homology searches or by synteny analysis, suggesting that this protein might play a significant role during the life cycle of *Plasmodium* but not for *Babesia* parasites. This may correlate with the observation that the *CCp* gene family encodes for proteins with parasite functions operating during sexual and other stages expressed in their arthropod vectors. Thus, the lack of *CCp4* genes might be related to the many differences that occur among the *Babesia* and *Plasmodium* life cycles, including the fact that *Babesia* parasites are transovarially transmitted, and the different nature of their arthropod hosts vectors in comparison with *Plasmodium* spp. (ticks *vs*. mosquitos). Alternatively, it is also possible that other *CCp genes* present in *Babesia* spp. play similar or overlapping functional roles as the *Plasmodium CCp4*.

CCp orthologs are also present in the rodent malaria species *Plasmodium berghei*, where they were termed LAP proteins. Several studies have been carried out to determine the function of this protein family in *P. berghei* (15, 32). Genetic crossover studies have shown that *Pb*CCp/LAP proteins are female-specific markers (33). In addition, recent studies on GFP fusions of *Pb*CCp3, *Pb*CCp1 and *Pb*CCp5 have shown protein expression in macrogametocytes and accumulation of these proteins in crystalloid organelles that form in the ookinete and persist until the early oocyst stage (34, 35). Crystalloids are transient specialized structures of malaria parasites which are present in mosquito-specific ookinetes and young oocyst stages of the parasite. They appear to play an important role in protein trafficking and sporozoite transmission and could be exploited as new targets for control of malaria transmission (36). Knockout of LAP1 (CCp3) or LAP3 (CCp5) was shown to completely abolish crystalloid formation in *P. berghei* (37). While sporozoites are formed in *Babesia* parasites, crystalloids and oocysts have not been so far reported, and whether CCp proteins may play similar roles in *Babesia spp*. remains unknown, and more research is needed.

The CCp5 and FNPA nucleotide and amino acid sequences were found to be highly conserved among otherwise distinct *B. bovis* isolates, and even among other related apicomplexans. High levels of sequence conservation among geographical strains is a very important criteria for selecting effective vaccine candidate antigens. Sequence comparisons of the *CCp5* and *FNPA* genes were also used to investigate whether immune selection pressure in the bovine host affects this gene family. The calculated ω (dN/dS) ratios for CCp5 and FNPA are <1, suggesting that these gene family members are unlikely exposed to host immune system selective forces.

In this study, we demonstrated transcription of the *CCp5* and *FNPA* genes in both blood and *in vitro* sexual stages of *B. bovis*. We also showed that the expression of *CCp5* and *FNPA* genes is up regulated in *B. bovis* sexual stages (Figure 4B). In addition, according to the RNAseq data, the *CCp5* and *FNPA* transcript levels in the kinete stage were found to be downregulated compared with blood stages. These results demonstrate that the expression of *CCp5* and *FNPA* genes needs to be tightly regulated and exert different transcriptional patterns at developmental stages of *B. bovis*, which may reflect distinct functional requirements. Furthermore, relatively low abundance of CCp5 and FNPA proteins were observed in both blood and sexual stages by immunoblot analysis. Consistently, *Pf* CCp5 and *Pf* FNPA have been reported to be less abundant in gametocytes than the other four *Pf* CCp proteins (31). In addition, *Pf* CCp5 also showed transcript expression in both asexual and sexual stage *Plasmodium* parasites (16). In this study, we demonstrated expression of CCp5 and FNPA proteins in blood and sexual stages by immunoblot and fixed IFA. Although a more intense fluorescent signal was observed in *in vitro* induced sexual stages than in blood stage parasites, we are limited to interpret this observation as a strong evidence of increased protein expression because sexual stage parasites tend to aggregate (38), which could result in more intense IFA signals and mislead the interpretation of the results.

Ideally, TBV candidate antigens should be widely conserved and expressed on the surface of the parasites, where they can be possible targets of antibodies. Bioinformatics analysis suggests that the CCp5 and FNPA proteins lack detectable signal peptides, but FNPA, unlike CCp5, has a transmembrane domain, suggesting that it might also be exposed on the surface of sexual stage parasites. Taking this data together, FNPA fulfills, at least in part, the criteria to be considered as a transmission blocking vaccine candidate.

Overall, the data in this study demonstrate the presence of *CCp5* and *FNPA* genes in *B. bovis* as non-canonical members of the CCp family, which might play important roles for the development of the parasite stages in ticks and vertebrates. Future studies need to be focused on knock out/knock in approaches of these genes in *B. bovis* for functional analyses.

## Conclusion

In this study, bioinformatics, pattern of expression, and localization analysis of *CCp5* and *FNPA* genes belonging to the *B. bovis* CCp family were performed. Findings from this study demonstrated expression of *CCp5* and *FNPA* genes and proteins in both blood and sexual stages of *B. bovis*, but their expression is increased in *in vitro* induced sexual stage parasites Furthermore, expression analysis, serology data, and the presence of a TM domain suggest that FNPA may be surface exposed during tick stages of the parasites, and thus, a possible candidate target for TBV.

## Data Availability Statement

The original contributions presented in the study are included in the article, further inquiries can be directed to the corresponding author/s.

## Author Contributions

CS, RB, and SO: conceptualization. SO, JL, and HA: investigation. SO, CS, HA, JL, and RB: formal analysis, writing, review, and editing. SO, HA, and CS: visualization. CS: supervision. CS and SO: funding acquisition. All authors contributed to the article and approved the submitted version.

## Funding

This work was supported by the Scientific and Technological Council of Turkey (TUBITAK) 2219 Grant Program, the International Development Research Center (IDRC) [Livestock Vaccine Innovation Fund (Grant 108525), funded by the Canadian Government and the Bill and Melinda Gates Foundation], the USDA CRIS Project 2090- 32000-039-00-D, and the USDA National Institute of Food and Agriculture (NIFA) (Award Number: 2020-67015-31809, Proposal Number: 2019-05375, Accession Number: 1022541).

## Conflict of Interest

The authors declare that the research was conducted in the absence of any commercial or financial relationships that could be construed as a potential conflict of interest.

## Publisher's Note

All claims expressed in this article are solely those of the authors and do not necessarily represent those of their affiliated organizations, or those of the publisher, the editors and the reviewers. Any product that may be evaluated in this article, or claim that may be made by its manufacturer, is not guaranteed or endorsed by the publisher.
