# Supplementary material for: Identification of CCp5 and FNPA as Novel Non-canonical Members of the CCp Protein Family in Babesia bovis
Source: Front Vet Sci. 2022 Feb 15;9:833183. doi: 10.3389/fvets.2022.833183 (PMC8886879; doi:10.3389/fvets.2022.833183)

Supplementary Material

**Supplementary Table 1.** Sequences of the primers used in this study.

| **Target** |  | **Primer sequence 5’-3’** |  | **Product size (bp)** |  | **References** |
| --- | --- | --- | --- | --- | --- | --- |
| *CCp5*-F |  | GAGCTCAAGGCAGAGATTAAGA |  | 104 |  | This study |
| *CCp5*-R |  | GTACCAAGTAAGCCGTCACTAA |  |  |  |  |
| *CCp5*-full F |  | ATGCGATGCAGGTTCATTCCTC |  | 3159 |  |  |
| *CCp5*-full R |  | TTACATCTCAGTGACCTTGACAAAT |  |  |  |  |
| *FNPA*-F |  | GTACCTGTTTCGGGAGACTATAC |  | 100 |  |  |
| *FNPA*-R |  | TACCCTCAAGTCCATGCTTAAT |  |  |  |  |
| *FNPA*-full F |  | ATGGCTACAATCCAAGGCGTTG |  | 2773 |  |  |
| *FNPA*-full R |  | CTACTTGAAGCAACTCACCCTATATA |  |  |  |  |
| *6CysA*-F |  | TGCTAGGGAATGTTGTAG |  | 137 |  |  |
| *6CysA*-R |  | CTCACCGTCCGGAACATAC |  |  |  |  |
| *Gapdh*-F |  | GTAGACTTCGTTGCTGAGTG |  | 104 |  |  |
| *Gapdh*-R |  | CTGGGAGGAGCAGAAATAATG |  |  |  |  |
| *RAP1*-F |  | CACGAGGAAGGAACTACCGATGTTGA |  | 354 |  | (30) |
| *RAP1*-R |  | CCAAGGAGCTTCAACGTACGAGGTCA |  |  |  |  |

**Supplementary Figure 1.** Schematic representation for the synteny map framework of *Pf*CCp4 gene with piroplasm species.**
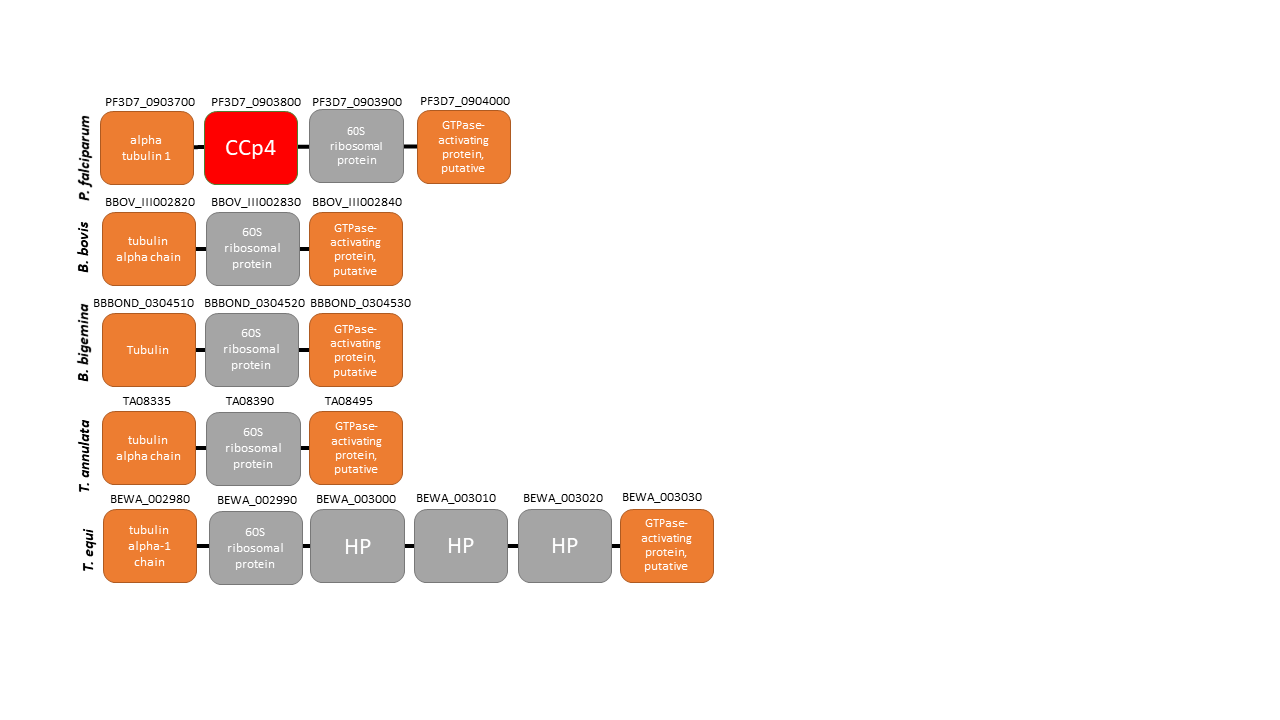
**

**Supplementary Figure 2.** Schematic domain architectures representation of piroplasm species of canonical members CCp5 and FNPA.**
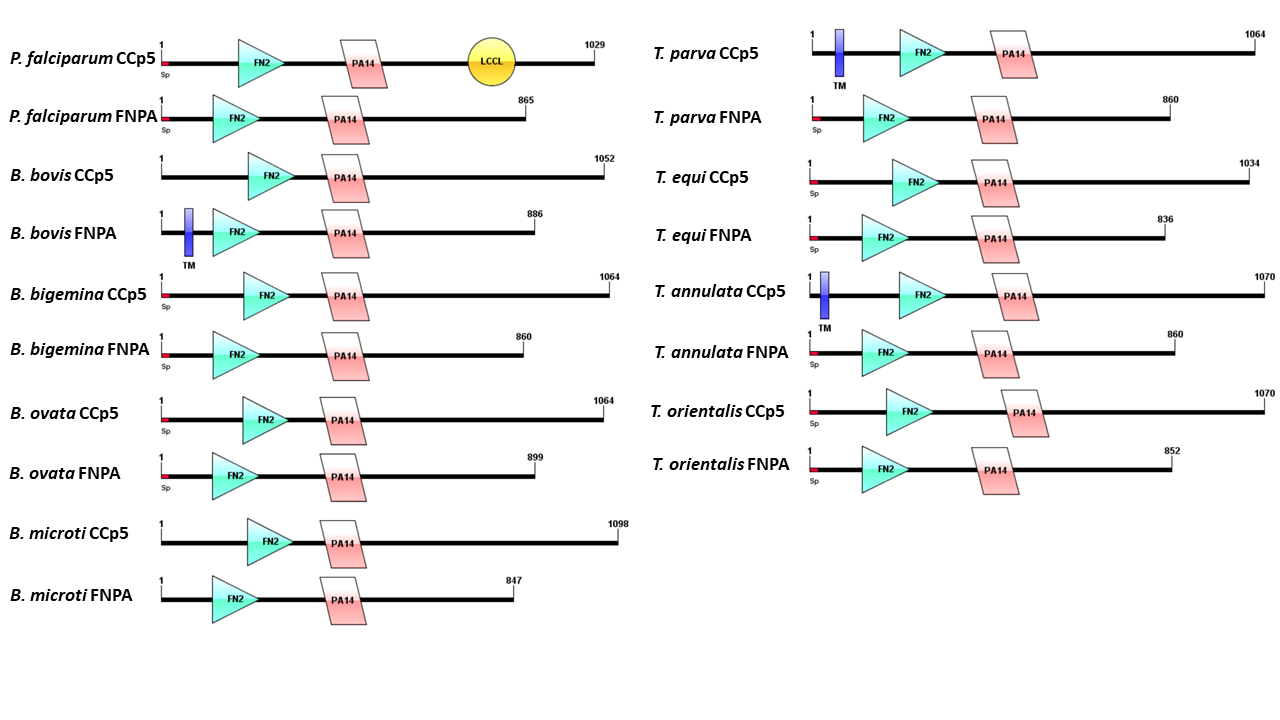
**

**Supplementary Figure 3.** Percentage of identity of nucleotide (a) and amino acid (b) of CCp5 and FNPA gene among different strains of *B. bovis*.

**
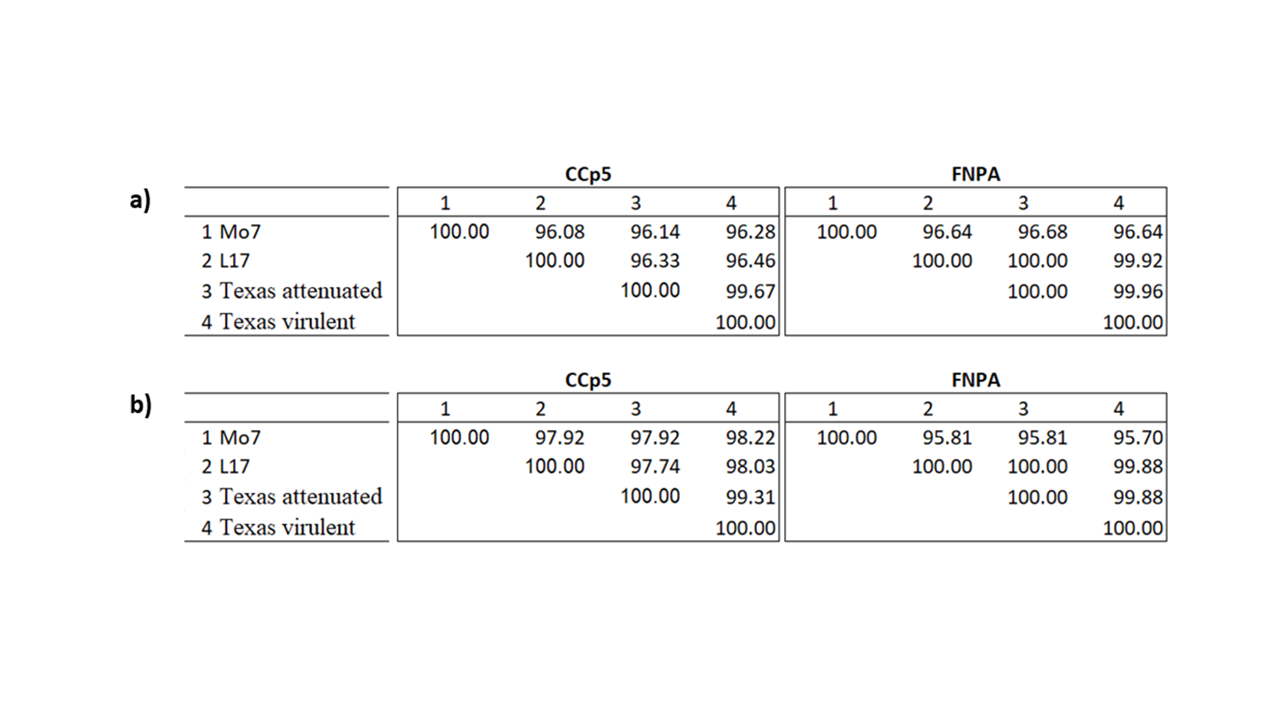
**

**Supplementary Figure 4.**

**A] Nucleotide sequences of *CCp5* and *FNPA* genes derived from different *B. bovis* strains.**

>ORF TxAt-CCp5

TGTGACAAATGCTTCCTTCAGTCTTTTTGAGGATCCCAGTGATTCTTCAAATAGTGACACATCGCAAACAGGTCTCTCTACGGATGCTAACAATGTTAGACAGGCCATTCCACCGGGAGCCGATGATGGCGTGGCTCCGCAATCAATCGCTACCTCTTCACAACCTCATGACATGAGTTCTTCTCTTATATCGCAACCTAATGCTATCCATGACCCTAAATTAGATCGTGTCATACCAATTCCAGGATCGGATTCTATAATTGGTGATGGATCTACTAATGAAGATGATAACGATTTAATGTACTATTTAACTCAGTATCGATCTCGTGTTCGTCGTACTGTTGAGGGTCATTTATGTGCTTCTGCTTTTGTTGAGAAGAACCAGATATACACCGATTGTACTATGGAGGTAGCTCCAGACGGTACTGTGGACCGTGAGTGGTGTTATTTGGAATCGCAGTTAAACGGTCAGTTAGAGCGTGATTGGGGGTTTTGTGTACCTCCATTGAATTATGAAAACATTCGTGGTGCCGTTTTAGGTAAGATTCGTGAGAAGGCTGACCAGGGCACTGAGTTAAAGCGTGCTTTACTGAAGCATAGGTCACTTTTATTAGGTATGGGCCGTCGTCTATCTATATACTGTGGCTCTGGCCACAAGGGCTTTGAACGATCATTAACACGTCTTAAGGAGATGGCTCAGAGTTCAGAGGACTCTCTGAAGGCTATGCGTTCGGTATCTAACGACGTGGTTGAGCTCAAGGCAGAGATTAAGAAGGACCAGGAACGTTACAATGCTCTGCGCCTTAAGATTCCTTCTGAGGTATTGACACAGGTTAGTGACGGCTTACTTGGTACTTATTACTCCGGTCATCTACTGGAATTTCCTGCGTATGGCAGTCGTATAGACCGTGAGTTGAACTTTGTCTTTTCTGACTTTATGCCTGTTGTTGGTTTGAATCCTCAGCGTTTCTCTGTTTTGTGGCACGGTTATATCCGTGCTCCTCATTCGGGTAACTTTGTTTTTAGTGTTACTACTAACAGTAACGTTCGTGTTGAATTGGATGGTGTGGAGATAATTAACAACGGACTAGTTCGTGAGGGTGACTCTGAATCCGGCTACCGTTATTTTATAAACCCTATGCTTCGTGGCGCTAGGCTAACATCGCAATCTCAGCAGCTTGTTGGAGGTCGTTTTTACAGGATTCAATTGGAATTCAGTCATTCTCAGCAGTATCACTATAGACCCAGGAATCCGTTCTCAAATTAGAGTGGTCCTCATTGAGACTGCCTCTTGAGGTAATAAAGGACTATTTATACACGAAAGCAGTCGCACCGCGTCTATCCATCAGTTATTTATCGCATGACCATTTCATTATTCACACTGCTATGAATGGAGTTTTGGCCTTTATGGATGATGACACAGCATTTTTGAATGATTTATCAAATGACCTCATAGGCACTACATTGTTGCGTACACTTCGATCACCAGGCTACCGGACGTTTGTATTACAACTTAATGTTACCTGTGCTATATACGTTGGATATATATCTGACGTGATACCCATAAGCACTGTGACTGAAGACGCCCTCGAGTTTATTGGATGTCCTGGATCATTAGTACATCTAGTTGATGGCACGGACGCCCCCAAACGTCTACATTTAAAGCGCGGTAAACTACAGCGTGGCCATACTTATACATTCCACGTGGAGGTAAAGAATTCACCGTTCTTTATATTCCTATCGATGCGTGACTTTTCTGAGCCTTTACAGGGTGAGGGTCCAATTAAGGTATTATCTATTCCTGACACCGAGTTATACCTCAGCTGTTCTGAGTCCTCTAGTATAGGTGATGGCTTCGGGTGCACTGCTGGTTTATCTGGTAAGCTACTTGACCAGAAACACGCCATTTGGCGTCCAACCGAAGGTCCTGGATCGTGGTTAAAGTTAGTATTCCGTCGTCCTGTACTGGTTACTGGATTCCAGATAAAGCAGAGCGACGACCCTTCTCGATGGGTTCGTCGGATATCACTTGACTACGATGACGGTTCTGAATTTTTCGAGCTTTTGCATTCAAATGACCCTAGGTCTAATTTATACCACCTAAATGAGCCTCGTTCATTGAATCAGGTCGTACTACGTATCGATGAGCTCTACACCAGTGCCCAGTTTACTGCCTTAGGTATTAATTTCCTGGGTCAGATGCTATCTGAGGAGCCAGCAGGACTTCGTCGCGTGCTAATGAATTGTCATGATACATTATCTGACAACATTGATGTCCAGCCTTTATCTGAGGGTCATTCGTACGAGCTTGTCTGTAGTCGTCGCTGCTTTGATGTATCATCTCCATTGGGCAAAGTCGAGGTACATGACATTTCTAAACCGCCGTGCGCCGCTCTTAATATTGACTTATGTCGTGCTGGTGGTGATCACTCAGTATGCCACGCTGCAATAACTATAATGAAGAAATCTGAACCCGGTACTGACGATGTATTTGGTTATGTGTTATCAAGGCTTGATCGTGGTGTTCGTGATGTACCTTTTCGTGCATCTATTTTGTTTAAAACGGGTACCAACAGATTCCCACAGCATAACTTCTTTGTGGACAACGGTTCTTTGAAGGGTCCTGTGGATACTTCTTATATTCGTCAAATTGGTGCCAACCCTCGTATGGGTGAGGTATCATACGGATGGCTTCGACCTAATGTAAGTGACGTCTACGAGCTGGGCATAGCATTGCCATTACCTGTGGAATCTCAGAAGTGCATTCAAACTCCTGGGTGCCAGCCTAACTTCTGGTCTATTGATTTACCTCGCAATGGCCGCTACAAGATCGAGTTAGTTTTGGGCAATTCGGTATCTTCGGATGTAACTACTGTGGACGACATAGTTCCCAATGGCGCTTTGGTTCCTCAGCGTCTGTGTTTGGAGGTTAATGGTCAGGTAGTCCTCAACTGTGTTGAGATTGCTGGCGGTTCACTTTACACCTTGGTCAAGGAGATTGAGGTGCGCAACAACTTGCTGAAGTTGACTTCTACTAGCAGCAACAGCTCG

>ORF TxVir-CCp5

TGTGACAAATGCTTCCTTCAGTCTTTTTGAGGATCCCAGTGATTCTTCAAATAGTGACACATCGCAAACAGGGCTCTCTACGGATGCTAACAATGTTAGACAGGCCATTCCACCGGGAGCCGATGATGGCGTGGCTCCGCAATCAATCGCTACCTCTTCACAACCTCATGACATGAGTTCTTCTCTTATATCGCAACCTAATGCTATCCATGACCCTAAATTAGATCGTGTCATACCAATTCCAGGATCGGATTCTATAATTGGTGATGGATCTACTAATGAAGATGATAACGATTTAATGTACTATTTAACTCAGTATCGATCTCGTGTTCGTCGTACTGTTGAGGGTCATTTATGTGCTTCTGCTTTTGTTGAGAAGAACCAGATATACACCGATTGTACTATGGAGGTAGCTCCAGACGGTACTGTGAACCGTGAGTGGTGTTATTTGGAATCGCAGTTAAACGGTCAGTTAGAGCGTGATTGGGGGTTTTGTGTACCTCCATTGAATTATGAAAACATTCGTGGTGCCGTTTTAGGTAAGATTCGTGAGAAGGCTGACCAGGGCACTGAGTTAAAGCGTGCTTTACTGAAGCATAGGTCACTTTTATTAGGTATGGGCCGTCGTCTATCTATATACTGTGGCTCTGGCCACAAGGGCTTTGAACGATCATTAACACGTCTTAAGGAGATGGTTCAGAGTTCAGAGGACTCTCTGAAGGCTATGCGTTCGGTATCTAACGACGTGGTTGAGCTCAAGGCAGAGATTAAGAAGGACCAGGAACGTTACAATGCTCTGCGCCTTAAGATTCCTTCTGAGGTATTGACCCAGGTTAGTGACGGCTTACTTGGTACTTATTACTCCGGTCATCTACTGGAATTTCCTGCGTATGGCAGTCGTATAGACCGTGAGTTGAACTTTGTCTTTTCTGACTTTATGCCTGTTGTTGGTTTGAATCCTCAGCGTTTCTCTGTTTTGTGGCACGGTTATATCCGTGCTCCTCATTCGGGTAACTTTGTTTTTAGTGTTACTACTAACAGTAACGTTCGTGTTGAATTGGATGGTGTGGAGATAATTAACAACGGACTAGTTCGTGAGGGTGACTCTGAATCCGGCTACCGTTATTTTATAAACCCTATGCTTCGTGGCGCTAGGCTAACATCGCAATCTCAGCAGCTTGTTGGAGGTCGTTTTTACAGGATTCAATTGGAATTCAGTCATTCTCAGCAGTATCACTATAGACCCAGTGAATCCGTTCTCAAATTAGAGTGGTCCTCATTGAGACTGCCTCTTGAGGTAATAAAGGACTATTTATACACGAAAGCAGTCGCACCGCGTCTATCCATCAGTTATTTATCGCATGACCATTTCATTATTCACACTGCTATGAATGGAGTTTTGGCCTTTATGGATGATGACACAGCATTTTTGAATGATTTATCAAATGACCTCATAGGCACTACATTGTTGCGTACACTTCGATCACCAGGCTACCGGACGTTTGTATTACAACTTAATGTTACCTGTGCTATATACGTTGGATATATATCTGACGTGATACCCATAAGCACTGTGACTGAAGACGCCCTCGAGTTTATTGGATGTCCTGGATCATTAGTACATCTAGTTGATGGCACGGACGCCCCCAAACGTCTACATTTAAAGCGCGGTAAACTACAGCGTGGCCATACTTATACATTCCACGTGGAGGTAAAGAATTCACCGTTCTTTATATTCCTATCGATGCGTGACTTTTCTGAGCCTTTACAGGGTGAGGGTCCAATTAAGGTATTATCTATTCCTGACACCGAGTTATACCTCAGCTGTTCTGAGTCCTCTAGTATAGGTGATGGCTTCGGGTGCACTGCTGGTTTATCTGGTAAGCTACTTGACCAGAAACACGCCATTTGGCGTCCAACCGAGGGTCCTGGATCGTGGTTAAAGTTAGTATTCCGTCGTCCTGTACTGGTTACTGGATTCCAGATAAAGCAGAGCGACGACCCTTCTCGATGGGTTCGTCGGATATCACTTGACTACGATGACGGTTCTGAATTTTTCGAGCTTTTGCATTCAAATGACCCTAGGTCTAATTTATACCACCTAAATGAGCCTCGTTCATTGAATCAGGTCATACTACGTATCGATGAGCTCTACACCAGTGCCCAGTCTACTGCCTTAGGTATTAATTTCCTGGGTCAGATGCTATCTGAGGAGTCAGCAGGACTTCGTCGCGTGCTAATGAATTGTCATGATACATTATCTGACAACATTGATGTCCAGCCTTTATCTGAGGGTCATTCGTACGAGCTTGTCTGTAGTCGTCGCTGCTTTGATGTATCATCTCCATTGGGCAAAGTCGAGGTACATGACATTTCTAAACCGCCGTGCGCCGCTCTTAATATTGACTTATGTCGTGCTGGTGGTGATCACTCAGTATGCCACGCTGCAATAACTATAATGAAGAAATCTGAACCCGGTACTGACGTTGTATTTGGTTATGTGTTATCAAGGCTTGATCGTGGTGTTCGTGATGTACCTTTTCGTGCATCTATTTTGTTTAAAACGGGTACCAACAGATTCCCACAGCATAACTTCTTTGTGGACAACGGTTCTTTGAAGGGTCCTGTGGATACTTCTTATATTCGTCAAATTGGTGCCAACCCTCGTATGGGTGAGGTATCATACGGATGGCTTCGACCTAATGTAAGTGGCGTCTACGAGCTGGGCATAGCATTGCCATTACCTGTGGAATCTCAGAAGTGCATTCAAACTCCTGGGTGCCAGCCTAACTTCTGGTCTATTGATTTACCTCGCAATGGCCGCTACAAGATCGAGTTAGTTTTGGGCAATTCGGTATCTTCGGATGTAACTACTGTGGACGACATAGTTCCCAATGGCGCTTTGGTTCCTCAGCGTCTGTGTTTGGAGGTTAATGGTCAGGTAGTCCTCAACTGTGTTGAGATTGCTGGCGGTTCACTTTACACCTTGGTCAAGGAGATTGAGGTGCGCAACAACTTGCTGAAGTTGACTTCTACTAGCAGCAACAGCTCG

>ORF L17-CCp5

TGTGACAAATGCTTCCTTCAGTCTTTTTGGGGATCCCTGTGATTCTTCAAATAGTGACACATCGCAAACAGGTCTCTCTACGGATGCTAGAAATGTTAGACAGGCCATTCCACCGGGAGCCGATGATGGCATGGCCCCGCAATCCATCGCTACCTCTTCACAACCTCATGACATGAGTTCTCCTATATCGCAACCTAATGCTATCCATGACCCTAAATTAGATCGTGTTATACCAATTCCCGGATCGGATTCTATAATTGGTGATGGAACTACTAATGAAGATGATAATGATTTAATGTACTATTTAACTCAGTATCGATCTCGTGTTCGTCGTACTGTAGAGGGTCATTTATGTGCTTCTGCTTTTGTTGAGAAGAATCAGATATACACTGATTGTACTATGGAGGTAGCTCCAGACGGTACTGTGAACCGTGAGTGGTGCTATTTGGAATCGCAGTTAAACGGCCAGTTAGAGCGTGATTGGGGGTTTTGTGTGCCTCCATTGAATTATGAAAACATTCGTGGTGCCGTTTTAGGTAAGATTCGTGAGAAGGCTGACCAGGGCACTGAGTTGAAGCGTGCATTACTGAAGCACAGGTCACTTTTATTAGGCATGGGTCGTCGTCTATCTATATACTGTGGCTCTGGCCACAAGGGCTTTGAACGATCTTTAACACGTCTTGAGGAGATGGTTCAGAGTTCAGAGAACTCTCTGAAGGCTATGCGTTCGGTATCTAACGACGTGGTTGAGCTCAAGGCAGAGATTAAGAAGGACCAGGAACGTTACAATGCTCTACGCCTTAAGATCCCTTCTGAGGTATTGACCCAGGTTAGTGACGGCTTACTTGGTACTTATTACTCTGGTCATCTACTGGAATTTCCTGCGTATGGCAGTCGTATAGACCGCGAGTTAAACTTTGTCTTTTCTGACTTTATGCCTGTTGTTGGTTTGAATCCTCAGCGTTTCTCTGTTTTGTGGCACGGTTATATCCGTGCTCCTCATTCGGGTAACTTTGTTTTTAGTGTTACTACTAACAGTTACGTTCGAGTTGAATTGGATGGTGTGGAGATAATTAACAACGGACTCGTTCGTGAGGGTGACTCTGAATCCGGCTACCGTTATTTTATAAACCCTATGCTTCGTGGCGCTAGGCTAACATCGCAATCTCAGCAGCTTGTTGGTGGTCGTTTTTACAGGATTCAATTGGAATTCAGCCATTCTCAGCAGTATCACTATAGACCCAGTGAATCCGTTCTCAAATTAGAGTGGTCCTCATTGAGACTGCCTCTTGAGGTAATAAAGGACTATTTATACACGAAAGCAGTCGCACCGCGTCTATCCATAAGTTATTTATCGCATGACCATTTCACTATTGACACTGCTATGAATGGAGTTTTGGCCTTTATGGATGATGACACAGCATTTTTGAACGACTTATCAAATGACCTTATAGGCACTACATTGTTGCGTACACTTCGATCACCTGGCTACCGGACGTTTGTATTACAACTTAATGTTACCTGTGGGATATATGTGGGATATATTTCTGACGTGATACCCATAAGCACTGTGACTGAGGACGCCCTCGAGTTTATTGGGTGTCCTGGGTCATTAGTACATCTCGTTGATGGTACTGATGCCCCCAAACGTCTACATTTAAAGCGTGGTAAACTACAGCGTGGCCATACTTATACATTCCACGTGGAGGTAAAGAATTCACCGTTCTTTATATTCCTATCGATGCGTGACTTTTCTGAGCCTTTACAGGGTGAGGGACCAATTAAGGTATTATCCATTCCAGACACCGAGTTATACCTCAGTTGTTCTGAGTCCTCTAGTATAGGTGATGGCTTTGGGTGCACTGCTGGTTTATCTGGGAAACTGCTTGACCAAAAACACGCCATTTGGCGTCCAACCGAGGGTCCTGGATCGTGGTTAAAGTTAGTATTCCGTCGTCCTGTACTGGTTACTGGATTCCAGATAAAGCAGAGTGACGACCCTTCTCGATGGGTTCGTCGGATATCACTTGACTACGATGACGGTACTGAATTTTTCGAGCTTTTGCATTCAAATGACCCTAGGTCTAATTTATATCACCTAAATGAGCCTCGTTCATTGAATCAGGTTATACTACGTATCGATGGGCTCTACACCAGTGCCCAGTCTACTGCCTTAGGTATTAATTTCCTGGGTCAGATGCTATCTGAGGAGTCAGCAGGACTTCGTCGCGTGCTAATGAACTGTCATGATACATTATCTGACAACGTTGATGTCCAGCCTTTATCTGAGGGTCATTCTTACGAGCTTGTTTGCAGTCGTCGCTGCTTTGATGTATCATCTCCATTGGGTAAGGTCGAGGTTCATGACATTTCTAAACCGCCGTGTACCGCGCTTAATATTGACTTATGTCGTGCTGGTGGCGATCACTCAGTATGTCACGCTGCAATAACTATAATGAAGAAATCGGAACCCGGTACTGACGATGTATTTGGTTATGTGTTATCAAGGCTTGATCGTGGTGTTCGTGATGTACCTTTCCGTGCATCTATTTTATTTAAGACGGGTACTAACAGATTCCCACAACACAACTTCTTTGTGGACAACGGTTCTTTGAAGGGTCCTGTGGATGTTTCTTATATTCGTCAAATTGGTGCCAACCCTCGTATGGGTGAGGTATCATACGGATGGCTTCGTCCTAATGTAAGTGACACCTACGAGCTGGGCATAGCATTGCCGTTACCTGTGGAATCTCAAAAATGCATTCAAACTCCTGGATGCCAGCCTAACTTCTGGTCTATTGATTTACCTCGCAATGGTCGATATAAGATCGAGTTAGTTTTGGGCAATTCGGTATCTTCGGATGTAACTACTGTGGATGACATAGTTCCCAATGGCGCCTTGGTTCCGCAGCGTCTGTGTTTGGAGGTTAATGGTCAGGTAGTCCTCAACTGTGTTGAGATTGCTGGCGGTTCACTTTACACCTTGGTCAAGGAGATTGAGGTGCGCAACAACTTGCTGAAGTTGACTTCTACTAGCAGCAACAGCTCG

>ORF Mo7-CCp5

TGTGACAAATGCTTCCTTCAGTCTTTTTGAGGATCCCAGTGATTCTTTAACTAGTGACACATCGCAAACAGGTCTCTCTACGGATGCTAACAATGTTAGACAGGCCATTCCACCGGGAGCCGATGATGGCACGGCTCCGCAATCAATCGCTACCTCTTCACAACCTCATGACATGAGTTCTCCTATATCGCAACCTAACGGTATCCATGACCCTAAATTAGATCGTGTTATACCAATTCCAGGATCGGATTCTATAATTGGTGATGGATCGACCAATGAAGATGATAATGATTTAATGTACTATTTAACTCAGTATCGATCTCGCGTTCGTCGTACTGTTGAGGGTCATTTATGTGCTTCTGCTTTTGTTGAGAAGAATCAGATATACACTGATTGTACTATGGAGGTAGCTCCAGACGGTACTGTTAACCGTGAGTGGTGTTATTTGGAATCGCAGTTAAACGGCCAGTTAGAGCGTGATTGGGGGTTTTGTGTACCTCCATTGAATTATGAGAACATTCGCGGTGCCGTTTTAGGTAAGATTCGTGAGAAGGCTGACCAGGGTACTGAGTTAAAGCGTGCTTTACTGAAGCATAGGTCACTTTTATTAGGAATGGGTCGTCGTCTATCTATATACTGTGGCTCTGGCCACAAGGGTTTTGAGCGATCCTTAACACGTCTTGAGGAGAGGGTTCAGAGTTCAGAGGACTCTCTGAAGGCTATGCGTTCGGTATCTAACGATGTGGTTGAGCTCAAGGCAGAGATTAAGAAGGACCAGGAACGTTACAATGCTCTGCGTCTTAAGATTCCTTCTGAGGTATTGACCCAGGTTAGTGACGGCTTACTTGGTACTTATTACTCCGGTCATCTACTTGAGTTTCCTGCGTATGGCAGTCGTATAGACCGTGAGTTGAACTTTGTCTTTTCTGACTTTATGCCTGTTGTTGGTTTGAATCCTCAGCGTTTCTCTGTTTTGTGGCACGGTTATATCCGTGCTCCTCATTCGGGTAACTTTGTTTTTAGTGTTACTACTAACAGTTACGTTCGAGTTGAATTGGATGGTGTGGAGATAATTAACAACGGACTAGTTCGTGAGGGTGACTCTGAATCCGGCTACCGTTATTTTATAAACCCTATGCTTCGTGGCGCTAGGCTAACATCGCAATCTCAGCAGCTTGTTGGAGGTCGTTTTTACAGGATTCAATTGGAATTCAGCCATTCTCAGCAGTATCACTATCGACCCAGTGAATCCGTTCTCAAATTAGAGTGGTCCTCTTTAAGGCTGCCTCTTGAGGTAATAAAGGACTATTTATACACTAAAGTGGTTGCACCGCGTCTATCCATAAGTTATTTATCGCATGACCATTTCATTATTGACACCGCTATGAATGGAGTTTTGGCCTTTATGGATGATGACACAGCATTTTTGAACGACTTATCAAATGACCTTATAGGCACTACATTGTTGCGTACACTTCGTTCACCAGGATACCGGACGTTTGTGCTACAGGTTAATGTTACTTGTGCTATATACGTTGGATATATATCTGACGTGATACCCATAAGCACTGTGACTGAAGATGCCCTTGAGTTTATTGGATGCCCTGGGTCATTAGTACACCTCGTTGATGGCACTGATGCCCCTAAACGTCTACATTTAAAGCGTGGTAAACTACAGCGTGGCCATACTTATACATTCCACGTGGTGGTAAAGAATTCACCGTTCTTTATATTCCTATCGATGCGTGACTTTGCCGAGCCTTTACAAGGTGAGGGACCAATTAAGGTACTATCTATTCCAGACACCGAGTTATACCTCAGCTGTTCTGAGTCCTCTAGTATAGGTGATGGCTTCGGGTGCACTGCTGGTTTATCTGGTAAACTGCTTGACCAGAAACACGCCATTTGGCGTCCAACTGAGGGCCCTGGATCATGGTTAAAGTTGGTATTCCGTCGTCCTGTTTTGGTTACTGGATTCCAGATAAAGCAGAGTGACGACCCTTCTCGATGGGTTCGTCGGATATCACTTGACTACGATGACGGTACTGAATTTTTCGAGCTTTTGCATTCAAATGACCCTAGGTCTAATTTATACCACCTAAATGAGCCTCGTTCATTGAATCAGGTTATACTACGTATCGATGAGCTCTACACCAGTGCCCAGTCTACTGCCTTGGGTATTAATTTCCTGGGTCAGATGCTATCTGAAGAGTCAGCAGGACTTCGTCGCGTATTAATGAATTGTCATGATACATTATCTGACAATATTGATGTCCAGCCTTTATCTGAGGGTCATTCTTACGAACTTGTTTGTAGTCGTCGCTGCTTTGATGTATCATCTCCAATGGGTAAAGTCGAGGTTCATGACATTTCTAAACCGCCGTGCGCCGCGCTTAATATTGACTTATGTCGTGCTGGTGGTGATCACTCAGTATGCCACGCTGCAATAACTATAATGAAGAAATCGGAACCCGGTACTGACGATGTATTTGGTTATGTGTTATCAAGGCTTGATCGTGGTGTTCGTGATGTACCTTTCCGTGCATCTATTTTGTTCAAGACGGGTACTAACAGATTCCCACAGCATAACTTCTTTGTGGACAACGGCTCTTTGAAGGGTCCTGTGGATGTTTCTTATATTCGTCAAATTGGTGCCAACCCTCGTATGGGTGAGGTATCATACGGATGGCTTCGTCCTAATGTAAGTGACGTCTACGAGCTGGGTATAGCATTGCCATTACCTGTGGAATCTCAAAAATGCATTCAAACTCCTGGGTGCCAGCCTAACTTCTGGTCTATTGATTTACCTCGCAATGGCCGCTATAAGATCGAGTTAGTTTTGGGCAATTCGGTATCTTCGGATGTAACTACTGTGGATGACATAGTTCCCAATGGCGCCTTGGTTCCGCAGCGTCTGTGTTTGGAGGTTAATGGTCAGGTAGTCCTCAACTGTGTTGAGATTGCTGGCGGTTCACTTTACACCTTGGTCAAGGAGATTGAGGTGCGCAACAACTTGCTGAAGTTGACTTCTACTAGCAGCAACAGCTCG

>ORF TxAt-FNPA

AAACCCTTTCGTATTATTCATTGCTAAGTGTGAGATTCTTATACTGCGTTTTCGTGTTTCATACTGTATTTGCCAATGGAGATATAAATCAGGATGTTGTTTCACCTAAGGATTTCACCATACCTTCAGGAACCGACTATGATCGGGAAGCCTTAATGGTAATTTGTATATACTTCACAATACTTATTTTACGTAGAAATTGACCGAATTTAGACAACGTCACAGGAAGACCGTTGATGGTATGCTTTGTGCAGCAGCTTTTGTGCGCAATGGGAATACATTTACGGACTGTACAACAACTGAAGCTCCAGATGGTTCTGTTGGTCGAGAATGGTGTTATGTAGAAGTACAGTTGATTGGTGTGGGCTCCAGAGATTGGGACTTTTGTGCCGGCACAGTAGACTATGATGTCATTCGATCCAAGGCATCTCTTTTATCGAAACTGAAATCTGAAGAGTTGGCACATTCAGTGTTACAACTTTCTGCAACTGAACAGTAGGTTATAATCTTTTTGTTAAAGATCCGCAGACGTTTGGAAAATACGCTAAAACATTTTGATGCTGTGTGTGGCCACGGAACGGCATCTCATGAAAATGATATGAACACAATATCGCACTCTCTCCGTGGTTTGGAGCGTGCTTTACAGCAGGTTGAGGCAAACTCACAAAGTCTCCACATGTTAAATGAGGAATACGAATCTCTGAGCGAGCAGCTTGAGATTACGCGTAAGAGCGTTTTAAATGACAAAAGAAATTGCTCAATTGTTCAAGGCTATTCCGTTGCGGTAGGTGATACATGGGTGGTAAATACATTATTGTTATATAGGTGGTGGGAGATGGTGTCCGTGCCAGCTACTTTGACAATCCATATCTAAGGGGGCCACCTGTTGGTTTCTTTGATCATTCTAGCCTATCTCTAACATTTGATGAGATATTACCTATAAATGGTGTAGACATTCGTTCCTTTTCCGTACGTTTTGAGACATATTTGCGCGTACCTGTTTCGGGAGACTATACCTTTTGGTTGGATGCTGATTGTAACTTTTTGATGTTCGTCGACGGAGAGTTGGTAATTAAGCATGGACTTGAGGGTAGGGGTACATTTGCATCGGCGTATAATTCAGCGGGTGTGGTTGCTCTTGACGGCCGTACAACAACCTCGACTCTCGTTTCTTCTCGCAAAATGTCACTGGTTGGGGGCAAGCGTTATCCATTGGTATTGGAATATTCACATCAAAGCTCACTGAAGTATCGTGATGAAAACGTTGTTCGTTTGATATTGAGTTGGGAAACTGACACGCGGTCCCGGACGATTATCGACCCGGATTATTTCTTCCGTAGCAGAGATTCTGGTGAATCTTTAATTATCAGTGGATTAGATGCGCGATGGTTTGACTTAGCCATGTTGGAGAACGGGGCACAGGCATTTATTAACGTAACAAATTTGTTGTTGGCAGACGTACCTGACCATCTCCGAGGCATGCGTATGGTACGCACAGTAGTGAAACCTGATTATGATACCGTGGATTTTTACATTTCGCATGATGCTTTTCTATATGTTGCTCAATCTGAACATCTGTCTTATATTCCTATGGCAGAGAATATGTCCACGTTCGATAGAAGCTGGGAGGTGATATCAGTTTATTCTATTGGCCACAATTGCGAAGAGGCCACCAGTCAACAGGAGTTTATAGTTTTCTACAAGCGATTCAAGGCAGGACCTGTAAAAATTCACATATTACCTGAAACGTCGTTTTTCTTGTTCATGCAGCCCGCTGCGGCTAATGCCATATGCCCCGACGATGTCCAGTATCTTCCTTTCAAAAATGGTGACGGTTGTGCGTCTTCTTCATCACTTTCAGCTGCATTTGACTGCTCAAAAGCTTTTGGGAGTGGATACTGGCAACCGAGTTCTGGGCGGATCACTGGACAGTGGCTGATGCGCACTTTCACCAACCCTGTGGAGTTGGTTCACTTCCATTTTTCTCCGATAGATGGCAGTTTACCTATGCGCGCAATTATATCATTTCCTGATGGATCTGAGGAAGATTTCGAGCTGCATTCACAGCTTCGTTACGAATTGGGATATCACGGCGTTGTGGATTCCATTAGGATTATGATTGAAACAATGAGCCCAGGAGACGGTATTACAGGTTCCGATACGGTTGATAATAAAGAAATAATTGGCGGAACATTCGCCTTTTATGGACGCGAATGCGGTGCTCGTACAACAGTGGAAGAAGCAGTACATTTTCCCATTCACATAAGTTTCTGCCAAGGGGGGCACGCATGTGGTCCTGATCACTTGGATTTGGGACACATGAAAGGATATCATGGCAGGTTGTCTTACGGTTGGGGTTCATCAAATGTCGTGGCTGATATAGCTGATCTTCAGATATGTAAACCCCAAGTGAATCACATAGTTGATGTTAACTTGGATTCTGTACCGTTGATAGATAGAGAATTAACAGAACTACCTGCGACGACTAAAAATCATAGTGTATATGATTTGTTATTGAAGCAGAAAGCTGGATTACCTCTTGGCCAGGGGCAACGGTGGACCATCGATGTCCCTGAGCATGGTGTATACAATGTTGAAGTTTTACTTTCTGCGTTATGTACTAATGTGGAATCTGCGTCCCTTCTCATAAACAAT

>ORF L17-FNPA

AAACCCTTTCGTATTATTCATTGCTAAGTGTGAGATTCTTATACTGCGTTTTCGTGTTTCATACTGTATTTGCCAATGGAGATATAAATCAGGATGTTGTTTCACCTAAGGATTTCACCATACCTTCAGGAACCGACTATGATCGGGAAGCCTTAATGGTAATTTGTATATACTTCACAATACTTATTTTACGTAGAAATTGACCGAATTTAGACAACGTCACAGGAAGACCGTTGATGGTATGCTTTGTGCAGCAGCTTTTGTGCGCAATGGGAATACATTTACGGACTGTACAACAACTGAAGCTCCAGATGGTTCTGTTGGTCGAGAATGGTGTTATGTAGAAGTACAGTTGATTGGTTGGGCTCCAGAGATTGGGACTTTTGTGCCGGCACAGTAGACTATGATGTCATTCGATCCAAGGCATCTCTTTTATCGAAACTGAAATCTGAAGAGTTGGCACATTCAGTGTTACAACTTTCTGCAACTGAACAGTAGGTTATAATCTTTTTGTTAAAGATCCGCAGACGTTTGGAAAATACGCTAAAACATTTTGATGCTGTGTGTGGCCACGGAACGGCATCTCATGAAAATGATATGAACACAATATCGCACTCTCTCCGTGGTTTGGAGCGTGCTTTACAGCAGGTTGAGGCAAACTCACAAAGTCTCCACATGTTAAATGAGGAATACGAATCTCTGAGCGAGCAGCTTGAGATTACGCGTAAGAGCGTTTTAAATGACAAAAGAAATTGCTCAATTGTTCAAGGCTATTCCGTTGCGGTAGGTGATACATGGGTGGTAAATACATTATTGTTATATAGGTGGTGGGAGATGGTGTCCGTGCCAGCTACTTTGACAATCCATATCTAAGGGGGCCACCTGTTGGTTTCTTTGATCATTCTAGCCTATCTCTAACATTTGATGAGATATTACCTATAAATGGTGTAGACATTCGTTCCTTTTCCGTACGTTTTGAGACATATTTGCGCGTACCTGTTTCGGGAGACTATACCTTTTGGTTGGATGCTGATTGTAACTTTTTGATGTTCGTCGACGGAGAGTTGGTAATTAAGCATGGACTTGAGGGTAGGGGTACATTTGCATCGGCGTATAATTCAGCGGGTGTGGTTGCTCTTGACGGCCGTACAACAACCTCGACTCTCGTTTCTTCTCGCAAAATGTCACTGGTTGGGGGCAAGCGTTATCCATTGGTATTGGAATATTCACATCAAAGCTCACTGAAGTATCGTGATGAAAACGTTGTTCGTTTGATATTGAGTTGGGAAACTGACACGCGGTCCCGGACGATTATCGACCCGGATTATTTCTTCCGTAGCAGAGATTCTGGTGAATCTTTAATTATCAGTGGATTAGATGCGCGATGGTTTGACTTAGCCATGTTGGAGAACGGGGCACAGGCATTTATTAACGTAACAAATTTGTTGTTGGCAGACGTACCTGACCATCTCCGAGGCATGCGTATGGTACGCACAGTAGTGAAACCTGATTATGATACCGTGGATTTTTACATTTCGCATGATGCTTTTCTATATGTTGCTCAATCTGAACATCTGTCTTATATTCCTATGGCAGAGAATATGTCCACGTTCGATAGAAGCTGGGAGGTGATATCAGTTTATTCTATTGGCCACAATTGCGAAGAGGCCACCAGTCAACAGGAGTTTATAGTTTTCTACAAGCGATTCAAGGCAGGACCTGTAAAAATTCACATATTACCTGAAACGTCGTTTTTCTTGTTCATGCAGCCCGCTGCGGCTAATGCCATATGCCCCGACGATGTCCAGTATCTTCCTTTCAAAAATGGTGACGGTTGTGCGTCTTCTTCATCACTTTCAGCTGCATTTGACTGCTCAAAAGCTTTTGGGAGTGGATACTGGCAACCGAGTTCTGGGCGGATCACTGGACAGTGGCTGATGCGCACTTTCACCAACCCTGTGGAGTTGGTTCACTTCCATTTTTCTCCGATAGATGGCAGTTTACCTATGCGCGCAATTATATCATTTCCTGATGGATCTGAGGAAGATTTCGAGCTGCATTCACAGCTTCGTTACGAATTGGGATATCACGGCGTTGTGGATTCCATTAGGATTATGATTGAAACAATGAGCCCAGGAGACGGTATTACAGGTTCCGATACGGTTGATAATAAAGAAATAATTGGCGGAACATTCGCCTTTTATGGACGCGAATGCGGTGCTCGTACAACAGTGGAAGAAGCAGTACATTTTCCCATTCACATAAGTTTCTGCCAAGGGGGGCACGCATGTGGTCCTGATCACTTGGATTTGGGACACATGAAAGGATATCATGGCAGGTTGTCTTACGGTTGGGGTTCATCAAATGTCGTGGCTGATATAGCTGATCTTCAGATATGTAAACCCCAAGTGAATCACATAGTTGATGTTAACTTGGATTCTGTACCGTTGATAGATAGAGAATTAACAGAACTACCTGCGACGACTAAAAATCATAGTGTATATGATTTGTTATTGAAGCAGAAAGCTGGATTACCTCTTGGCCAGGGGCAACGGTGGACCATCTATGTCCCTGAGCATGGTGTATACAATGTTGAAGTTTTACTTTCTGCGTTATGTACTAATGTGGAATCTGCGTCCCTTCTCATAAACAATG

>ORF Mo7-FNPA

AAACCCTTTTGTATTACTCATTGCTAAGTGTGAGATTCTTATACTGCGTGTTCGTGTTTCATACTGTATTTGCCAATGGAGATATAAATAAGGATGTTGTTTCACCTAAGGATTTCACCATACCTTCAGGAACCGACTATGATCGGGAAGCCTTAATGGTAATTTGTATATACTTCATAACACTTATTTTACGTAGAAATTGACCGAATTTAGACAACGTCACAGGAAGACCGTTGATGGTATGCTTTGTGCAGCAGCTTTTGTGCGCAATGGGAATACATTTACGGACTGTACAACAACTGAAGCTCCAGATGGTTCTGTCGGTAGAGAATGGTGTTATGTAGAAGTACAGTTGATTGGTGTGGGCTCCAGGGATTGGGACTTTTGTGCCGGCACAGTAGACTATGATGTCATTCGATCCAAGGCATCACTTTTATCGAAACTGAAATCCGATGAGTTGGGACATTCCGTGTTACAACTTTCTGCAACTGAACAGTAGGTTATAATCTTTTTGTTAAAGATCCACAGACGTTTGGAAAATACGCTCAAACATTTTGATGCGGTCTGTGGTCACGGAACGGCATCTCACGAAAATGATATGAACACAATATCGCATTCTCTCCGTGGTTTGGAGCGTGCTTTACAGCAGGTTGAGGCAAACTCACAAAGTCTCCACATGTTAAGTGAGGAATACGAATCTCTGAGCGAGCAGCTTGAGATTACGCGTAAGAGCGTTTTAAATGACAAAAGAAATTGCTCAATTGTGCAAGGCTATTCCGCTGCGGTAGGTGATACATGGGTGGTAAATTCATTATTGGTATATAGGTGGTGGGAGATGGCGTCCGTGCCAGCTACTTTGACAATCCATATCTAAGGGGACCACCTGTTGGTTTCTTTGATCATTCTAGCCTATCTCTAACATTTGATGAGATATTACCAATAAATGGTGTAGACATTCGTTCCTTTTCCGTACGTTTTGAGACATATTTGCGCGTACCTGTTTCGGGAGACTATACCTTTTGGTTGGATGCTGATTGTAACTTTTTGATGTTCGTAGACGGAGAGTTGGTAATTAAGCATGGACTTGAGGGTAGGGGTACATTTGCGTCGGCGTATAATTCAACGGGTGTGGTTGCTCTTGACGGCCGTACAACAACCTCGACTCTCGTTTCTTCTCGCAAAATGTCACTGGTTGGGGGCAAGCGTTATCCATTGGTTTTGGAGTATTCACATCAAAGCTCACTGAAGTATCGTGATGAAAACGTTGGTCGTTTAGTATTGAGTTGGGAAACTGACATGCGGTCCCGGACGATTATCGACCCGGACTATTTCTTCCGTAGTAGAGATTCTGGTGAATCTTTAATTATCAGTGGATTAGATGCGCGATGGTTTGACTTAGCCATGTTGGAGAACGGGGCACAGGCATTCATTAACGTGACCAATTTGTTGTTGGCAGACGTACCTGACCATCTCCGAGGCATGCGTATGGTACGCACAGTAGTGAAACCTGATTATGATACCGTGGATTTTTACATTTCCCATGATGCTTTTCTATATGTTGCTCAATCTGAACATCTGTCTTATATTCCTATGGCAGAGAATATGTCTATGTTCGATCGAAGCTGGGAGGTGATATCAGTATATTCTATTGGCCACAATTGCGAAGAGGCCATCAGTCAACAGGAGTTTATAATTTTCTACAAGCGATTTAAGGCAGGACCTGTAAAAATCCACATATTACCCGAAACGTCGTTTTTCTTGTTCATGCAGCCCGCTGCAGCTAATGCAATATGTCCCGACGATGTCCAGTATCTTCCTTTCAAAAACGGTGACGGTTGTGCGTCTTCTTCATCACTTTCAGCCGCATTTGACTGCTCAAAAGCATTTGGGAGTGGTTACTGGCAACCGAGTTCTGGGCGGATCACTGGACAGTGGCTGATGCGCACTTTCACCAACCCTGTGGAGTTGGTTCACTTCCATTTTTCTCCGATATATGGTGGTTTACCTATGCACGCAATTATATCATTTCCCGATGGATCTGAGGAAGATTTCGAGCTGCATTCACAGCTTCGTTACGAATTGGGATACCACGGCGTTGTGGATTCCATTAGGATCATGATTGAAACAATGAGCCCAGGAGATGGCATTACAGGTTCCGATACGGTTGATAATAAAGAAATAATTGGCGGAACATTCGCCTTTTATGGACGCGAATGCGGCGCTCGTACAACAGTGGAAGAAGCAGTACATTTTCCCATTCACATAAGTTTCTGCCAAGGGGGACACGCTTGTGGTCCTGATCACTTGGATTTGGGACACACGAAAGGATATCATGGCAGGTTGTCTTACGGTTGGGCTTCATCAAATGTCGTTGCTGATATTGCTGATATTCAGATATGTAGACCTCAAGTGAATCACATAGTTGATGTTAACTTGGATTCTGTACCGTTGATAGATAGAGAATTAACAGAACTATCTGCAACGACTAAAAAGCATAGTGTATATGAATTGTTATTGAAGCAGAAAGCTGGACTACCTCTTGGCCAGGGGCAACGGTGGACCATCGACGTCCCTGAGCATGGTGTATACAAAGTTGAAGTTTTACTTTCTGCGTTATGTACTAATGTGGATTCTGCGTCTCTTCTCATAAA

>ORF TxVir2-FNPA

AAACCCTTTCGTATTATTCATTGCTAAGTGTGAGATTCTTATACTGCGTTTTCGTGTTTCATACTGTATTTGCCAATGGAGATATAAATCAGGATGTTGTTTCACCTAAGGATTTCACCATACCTTCAGGAACCGACTATGATCGGGAAGCCTTAATGGTAATTTGTATATACTTCACAATACTTATTTTACGTAGAAATTGACCGAATTTAGACAACGTCACAGGAAGACCGTTGATGGTATGCTTTGTGCAGCAGCTTTTGTGCGCAATGGGAATACATTTACGGACTGTACAACAACTGAAGCTCCAGATGGTTCTGTTGGTCGAGAATGGTGTTATGTAGAAGTACAGTTGATTGGTGTGGGCTCCAGAGATTGGGACTTTTGTGCCGGCACAGTAGACTATGATGTCATTCGATCCAAGGCATCTCTTTTATCGAAACTGAAATCTGAAGAGTTGGCACATTCAGTGTTACAACTTTCTGCAACTGAACAGTAGGTTATAATCTTTTTGTTAAAGATCCGCAGACGTTTGGAAAATACGCTAAAACATTTTGATGCTGTGTGTGGCCACGGAACGGCATCTCATGAAAATGATATGAACACAATATCGCACTCTCTCCGTGGTTTGGAGCGTGCTTTACAGCAGGTTGAGGCAAACTCACAAAGTCTCCACATGTTAAATGAGGAATACGAATCTCTGAGCGAGCAGCTTGAGATTACGCGTAAGAGCGTTTTAAATGACAAAAGAAATTGCTCAATTGTTCAAGGCTATTCCGTTGCGGTAGGTGATACATGGGTGGTAAATACATTATTGTTATATAGGTGGNGGGAGATGGTGTCCGTGCCAGCTACTTTGACAATCCATATCTAAGGGGGCCACCTGTTGGTTTCTTTGATCATTCTAGCCTATCTCTAACATTTGATGAGATATTACCTATAAATGGTGTAGACATTCGTTCCTTTTCCGTACGTTTTGAGACATATTTGCGCGTACCTGTTTCGGGAGACTATACCTTTTGGTTGGATGCTGATTGTAACTTTTTGATGTTCGTCGACGGAGAGTTGGTAATTAAGCATGGACTTGAGGGTAGGGGTACATTTGCATCGGCGTATAATTCAGCGGGTGTGGTTGCTCTTGACGGCCGTACAACAACCTCGACTCTCGTTTCTTCTCGCAAAATGTCACTGGTTGGGGGCAAGCGTTATCCATTGGTATTGGAATATTCACATCAAAGCTCACTGAAGTATCGTGATGAAAACGTTGTTCGTTTGATATTGAGTTGGGAAACTGACACGCGGTCCCGGACGATTATCGACCCGGATTATTTCTTCCGTAGCAGAGATTCTGGTGAATCTTTAATTATCAGTGGATTAGATGCGCGATGGTTTGACTTAGCCATGTTGGAGAACGGGGCACAGGCATTTATTAACGTAACAAATTTGTTGTTGGCAGACGTACCTGACCATCTCCGAGGCATGCGTATGGTACGCACAGTAGTGAAACCTGATTATGATACCGTGGATTTTTACATTTCGCATGATGCTTTTCTATATGTTGCTCAATCTGAACATCTGTCTTATATTCCTATGGCAGAGAATATGTCCACGTTCGATAGAAGCTGGGAGGTGATATCAGTTTATTCTATTGGCCACAATTGCGAAGAGGCCACCAGTCAACAGGAGTTTATAGTTTTCTACAAGCGATTCAAGGCAGGACCTGTAAAAATTCACATATTACCTGAAACGTCGTTTTTCTTGTTCATGCAGCCCGCTGCGGCTAATGCCATATGCCCCGACGATGTCCAGTATCTTCCTTTCAAAAATGGTGACGGTTGTGCGTCTTCTTCATCACTTTCAGCTGCATTTGACTGCTCAAAAGCTTTTGGGAGTGGATACTGGCAACCGAGTTCTGGGCGGATCACTGGACAGTGGCTGATGCGCACTTTCACCAACCCTGTGGAGTTGGTTCACTTCCATTTTTCTCCGATAGATGGCAGTTTACCTATGCGCGCAATTATATCATTTCCTGATGGATCTGAGGAAGATTTCGAGCTGCATTCACAGCTTCGTTACGAATTGGGATATCACGGCGTTGTGGATTCCATTAGGATTATGATTGAAACAATGAGCCCAGGAGACGGTATTACAGGTTCCGATACGGTTGATAATAAAGAAATAATTGGCGGAACATTCGCCTTTTATGGACGCGAATGCGGTGCTCGTACAACAGTGGAAGAAGCAGTACATTTTCCCATTCACATAAGTTTCTGCCAAGGGGGGCACGCATGTGGTCCTGATCACTTGGATTTGGGACACATGAAAGGATATCATGGCAGGTTGTCTTACGGTTGGGGTTCATCAAATGTCGTGGCTGATATAGCTGATCTTCAGATATGTAAACCCCAAGTGAATCACATAGTTGATGTTAACTTGGATTCTGTACCGTTGATAGATAGAGAATTAACAGAACTACCTGCGACGACTAAAAATCATAGTGTATATGATTTGTTATTGAAGCAGAAAGCTGGATTACCTCTTGGCCAGGGGCAACGGTGGACCATCGATGTCCCTGAGCATGGTGTATACAATGTTGAAGTTTTACTTTCTGCGTTATGTACTAATGTGGAATCTGCGTCCCTTCTCATAAACAA

**[B]**

**Multiple sequence alignment of *CCp5* and *FNPA* derived from distinct *B. bovis* strains**

Mo7-CCp5 TGTGACAAATGCTTCCTTCAGTCTTTTTGAGGATCCCAGTGATTCTTTAACTAGTGACAC

L17-CCp5 TGTGACAAATGCTTCCTTCAGTCTTTTTGGGGATCCCTGTGATTCTTCAAATAGTGACAC

TxAt-CCp5 TGTGACAAATGCTTCCTTCAGTCTTTTTGAGGATCCCAGTGATTCTTCAAATAGTGACAC

TxVir-CCp5 TGTGACAAATGCTTCCTTCAGTCTTTTTGAGGATCCCAGTGATTCTTCAAATAGTGACAC

***************************** ******* ********* ** *********

Mo7-CCp5 ATCGCAAACAGGTCTCTCTACGGATGCTAACAATGTTAGACAGGCCATTCCACCGGGAGC

L17-CCp5 ATCGCAAACAGGTCTCTCTACGGATGCTAGAAATGTTAGACAGGCCATTCCACCGGGAGC

TxAt-CCp5 ATCGCAAACAGGTCTCTCTACGGATGCTAACAATGTTAGACAGGCCATTCCACCGGGAGC

TxVir-CCp5 ATCGCAAACAGGGCTCTCTACGGATGCTAACAATGTTAGACAGGCCATTCCACCGGGAGC

************ **************** *****************************

Mo7-CCp5 CGATGATGGCACGGCTCCGCAATCAATCGCTACCTCTTCACAACCTCATGACATGAG---

L17-CCp5 CGATGATGGCATGGCCCCGCAATCCATCGCTACCTCTTCACAACCTCATGACATGAG---

TxAt-CCp5 CGATGATGGCGTGGCTCCGCAATCAATCGCTACCTCTTCACAACCTCATGACATGAGTTC

TxVir-CCp5 CGATGATGGCGTGGCTCCGCAATCAATCGCTACCTCTTCACAACCTCATGACATGAGTTC

********** *** ******** ********************************

Mo7-CCp5 TTCTCCTATATCGCAACCTAACGGTATCCATGACCCTAAATTAGATCGTGTTATACCAAT

L17-CCp5 TTCTCCTATATCGCAACCTAATGCTATCCATGACCCTAAATTAGATCGTGTTATACCAAT

TxAt-CCp5 TTCTCTTATATCGCAACCTAATGCTATCCATGACCCTAAATTAGATCGTGTCATACCAAT

TxVir-CCp5 TTCTCTTATATCGCAACCTAATGCTATCCATGACCCTAAATTAGATCGTGTCATACCAAT

***** *************** * *************************** ********

Mo7-CCp5 TCCAGGATCGGATTCTATAATTGGTGATGGATCGACCAATGAAGATGATAATGATTTAAT

L17-CCp5 TCCCGGATCGGATTCTATAATTGGTGATGGAACTACTAATGAAGATGATAATGATTTAAT

TxAt-CCp5 TCCAGGATCGGATTCTATAATTGGTGATGGATCTACTAATGAAGATGATAACGATTTAAT

TxVir-CCp5 TCCAGGATCGGATTCTATAATTGGTGATGGATCTACTAATGAAGATGATAACGATTTAAT

*** *************************** * ** ************** ********

Mo7-CCp5 GTACTATTTAACTCAGTATCGATCTCGCGTTCGTCGTACTGTTGAGGGTCATTTATGTGC

L17-CCp5 GTACTATTTAACTCAGTATCGATCTCGTGTTCGTCGTACTGTAGAGGGTCATTTATGTGC

TxAt-CCp5 GTACTATTTAACTCAGTATCGATCTCGTGTTCGTCGTACTGTTGAGGGTCATTTATGTGC

TxVir-CCp5 GTACTATTTAACTCAGTATCGATCTCGTGTTCGTCGTACTGTTGAGGGTCATTTATGTGC

*************************** ************** *****************

Mo7-CCp5 TTCTGCTTTTGTTGAGAAGAATCAGATATACACTGATTGTACTATGGAGGTAGCTCCAGA

L17-CCp5 TTCTGCTTTTGTTGAGAAGAATCAGATATACACTGATTGTACTATGGAGGTAGCTCCAGA

TxAt-CCp5 TTCTGCTTTTGTTGAGAAGAACCAGATATACACCGATTGTACTATGGAGGTAGCTCCAGA

TxVir-CCp5 TTCTGCTTTTGTTGAGAAGAACCAGATATACACCGATTGTACTATGGAGGTAGCTCCAGA

********************* *********** **************************

Mo7-CCp5 CGGTACTGTTAACCGTGAGTGGTGTTATTTGGAATCGCAGTTAAACGGCCAGTTAGAGCG

L17-CCp5 CGGTACTGTGAACCGTGAGTGGTGCTATTTGGAATCGCAGTTAAACGGCCAGTTAGAGCG

TxAt-CCp5 CGGTACTGTGGACCGTGAGTGGTGTTATTTGGAATCGCAGTTAAACGGTCAGTTAGAGCG

TxVir-CCp5 CGGTACTGTGAACCGTGAGTGGTGTTATTTGGAATCGCAGTTAAACGGTCAGTTAGAGCG

********* ************* *********************** ***********

Mo7-CCp5 TGATTGGGGGTTTTGTGTACCTCCATTGAATTATGAGAACATTCGCGGTGCCGTTTTAGG

L17-CCp5 TGATTGGGGGTTTTGTGTGCCTCCATTGAATTATGAAAACATTCGTGGTGCCGTTTTAGG

TxAt-CCp5 TGATTGGGGGTTTTGTGTACCTCCATTGAATTATGAAAACATTCGTGGTGCCGTTTTAGG

TxVir-CCp5 TGATTGGGGGTTTTGTGTACCTCCATTGAATTATGAAAACATTCGTGGTGCCGTTTTAGG

****************** ***************** ******** **************

Mo7-CCp5 TAAGATTCGTGAGAAGGCTGACCAGGGTACTGAGTTAAAGCGTGCTTTACTGAAGCATAG

L17-CCp5 TAAGATTCGTGAGAAGGCTGACCAGGGCACTGAGTTGAAGCGTGCATTACTGAAGCACAG

TxAt-CCp5 TAAGATTCGTGAGAAGGCTGACCAGGGCACTGAGTTAAAGCGTGCTTTACTGAAGCATAG

TxVir-CCp5 TAAGATTCGTGAGAAGGCTGACCAGGGCACTGAGTTAAAGCGTGCTTTACTGAAGCATAG

*************************** ******** ******** *********** **

Mo7-CCp5 GTCACTTTTATTAGGAATGGGTCGTCGTCTATCTATATACTGTGGCTCTGGCCACAAGGG

L17-CCp5 GTCACTTTTATTAGGCATGGGTCGTCGTCTATCTATATACTGTGGCTCTGGCCACAAGGG

TxAt-CCp5 GTCACTTTTATTAGGTATGGGCCGTCGTCTATCTATATACTGTGGCTCTGGCCACAAGGG

TxVir-CCp5 GTCACTTTTATTAGGTATGGGCCGTCGTCTATCTATATACTGTGGCTCTGGCCACAAGGG

*************** ***** **************************************

Mo7-CCp5 TTTTGAGCGATCCTTAACACGTCTTGAGGAGAGGGTTCAGAGTTCAGAGGACTCTCTGAA

L17-CCp5 CTTTGAACGATCTTTAACACGTCTTGAGGAGATGGTTCAGAGTTCAGAGAACTCTCTGAA

TxAt-CCp5 CTTTGAACGATCATTAACACGTCTTAAGGAGATGGCTCAGAGTTCAGAGGACTCTCTGAA

TxVir-CCp5 CTTTGAACGATCATTAACACGTCTTAAGGAGATGGTTCAGAGTTCAGAGGACTCTCTGAA

***** ***** ************ ****** ** ************* **********

Mo7-CCp5 GGCTATGCGTTCGGTATCTAACGATGTGGTTGAGCTCAAGGCAGAGATTAAGAAGGACCA

L17-CCp5 GGCTATGCGTTCGGTATCTAACGACGTGGTTGAGCTCAAGGCAGAGATTAAGAAGGACCA

TxAt-CCp5 GGCTATGCGTTCGGTATCTAACGACGTGGTTGAGCTCAAGGCAGAGATTAAGAAGGACCA

TxVir-CCp5 GGCTATGCGTTCGGTATCTAACGACGTGGTTGAGCTCAAGGCAGAGATTAAGAAGGACCA

************************ ***********************************

Mo7-CCp5 GGAACGTTACAATGCTCTGCGTCTTAAGATTCCTTCTGAGGTATTGACCCAGGTTAGTGA

L17-CCp5 GGAACGTTACAATGCTCTACGCCTTAAGATCCCTTCTGAGGTATTGACCCAGGTTAGTGA

TxAt-CCp5 GGAACGTTACAATGCTCTGCGCCTTAAGATTCCTTCTGAGGTATTGACACAGGTTAGTGA

TxVir-CCp5 GGAACGTTACAATGCTCTGCGCCTTAAGATTCCTTCTGAGGTATTGACCCAGGTTAGTGA

****************** ** ******** ***************** ***********

Mo7-CCp5 CGGCTTACTTGGTACTTATTACTCCGGTCATCTACTTGAGTTTCCTGCGTATGGCAGTCG

L17-CCp5 CGGCTTACTTGGTACTTATTACTCTGGTCATCTACTGGAATTTCCTGCGTATGGCAGTCG

TxAt-CCp5 CGGCTTACTTGGTACTTATTACTCCGGTCATCTACTGGAATTTCCTGCGTATGGCAGTCG

TxVir-CCp5 CGGCTTACTTGGTACTTATTACTCCGGTCATCTACTGGAATTTCCTGCGTATGGCAGTCG

************************ *********** ** ********************

Mo7-CCp5 TATAGACCGTGAGTTGAACTTTGTCTTTTCTGACTTTATGCCTGTTGTTGGTTTGAATCC

L17-CCp5 TATAGACCGCGAGTTAAACTTTGTCTTTTCTGACTTTATGCCTGTTGTTGGTTTGAATCC

TxAt-CCp5 TATAGACCGTGAGTTGAACTTTGTCTTTTCTGACTTTATGCCTGTTGTTGGTTTGAATCC

TxVir-CCp5 TATAGACCGTGAGTTGAACTTTGTCTTTTCTGACTTTATGCCTGTTGTTGGTTTGAATCC

********* ***** ********************************************

Mo7-CCp5 TCAGCGTTTCTCTGTTTTGTGGCACGGTTATATCCGTGCTCCTCATTCGGGTAACTTTGT

L17-CCp5 TCAGCGTTTCTCTGTTTTGTGGCACGGTTATATCCGTGCTCCTCATTCGGGTAACTTTGT

TxAt-CCp5 TCAGCGTTTCTCTGTTTTGTGGCACGGTTATATCCGTGCTCCTCATTCGGGTAACTTTGT

TxVir-CCp5 TCAGCGTTTCTCTGTTTTGTGGCACGGTTATATCCGTGCTCCTCATTCGGGTAACTTTGT

************************************************************

Mo7-CCp5 TTTTAGTGTTACTACTAACAGTTACGTTCGAGTTGAATTGGATGGTGTGGAGATAATTAA

L17-CCp5 TTTTAGTGTTACTACTAACAGTTACGTTCGAGTTGAATTGGATGGTGTGGAGATAATTAA

TxAt-CCp5 TTTTAGTGTTACTACTAACAGTAACGTTCGTGTTGAATTGGATGGTGTGGAGATAATTAA

TxVir-CCp5 TTTTAGTGTTACTACTAACAGTAACGTTCGTGTTGAATTGGATGGTGTGGAGATAATTAA

********************** ******* *****************************

Mo7-CCp5 CAACGGACTAGTTCGTGAGGGTGACTCTGAATCCGGCTACCGTTATTTTATAAACCCTAT

L17-CCp5 CAACGGACTCGTTCGTGAGGGTGACTCTGAATCCGGCTACCGTTATTTTATAAACCCTAT

TxAt-CCp5 CAACGGACTAGTTCGTGAGGGTGACTCTGAATCCGGCTACCGTTATTTTATAAACCCTAT

TxVir-CCp5 CAACGGACTAGTTCGTGAGGGTGACTCTGAATCCGGCTACCGTTATTTTATAAACCCTAT

********* **************************************************

Mo7-CCp5 GCTTCGTGGCGCTAGGCTAACATCGCAATCTCAGCAGCTTGTTGGAGGTCGTTTTTACAG

L17-CCp5 GCTTCGTGGCGCTAGGCTAACATCGCAATCTCAGCAGCTTGTTGGTGGTCGTTTTTACAG

TxAt-CCp5 GCTTCGTGGCGCTAGGCTAACATCGCAATCTCAGCAGCTTGTTGGAGGTCGTTTTTACAG

TxVir-CCp5 GCTTCGTGGCGCTAGGCTAACATCGCAATCTCAGCAGCTTGTTGGAGGTCGTTTTTACAG

********************************************* **************

Mo7-CCp5 GATTCAATTGGAATTCAGCCATTCTCAGCAGTATCACTATCGACCCAGTGAATCCGTTCT

L17-CCp5 GATTCAATTGGAATTCAGCCATTCTCAGCAGTATCACTATAGACCCAGTGAATCCGTTCT

TxAt-CCp5 GATTCAATTGGAATTCAGTCATTCTCAGCAGTATCACTATAGACCCAG-GAATCCGTTCT

TxVir-CCp5 GATTCAATTGGAATTCAGTCATTCTCAGCAGTATCACTATAGACCCAGTGAATCCGTTCT

****************** ********************* ******* ***********

Mo7-CCp5 CAAATTAGAGTGGTCCTCTTTAAGGCTGCCTCTTGAGGTAATAAAGGACTATTTATACAC

L17-CCp5 CAAATTAGAGTGGTCCTCATTGAGACTGCCTCTTGAGGTAATAAAGGACTATTTATACAC

TxAt-CCp5 CAAATTAGAGTGGTCCTCATTGAGACTGCCTCTTGAGGTAATAAAGGACTATTTATACAC

TxVir-CCp5 CAAATTAGAGTGGTCCTCATTGAGACTGCCTCTTGAGGTAATAAAGGACTATTTATACAC

****************** ** ** ***********************************

Mo7-CCp5 TAAAGTGGTTGCACCGCGTCTATCCATAAGTTATTTATCGCATGACCATTTCATTATTGA

L17-CCp5 GAAAGCAGTCGCACCGCGTCTATCCATAAGTTATTTATCGCATGACCATTTCACTATTGA

TxAt-CCp5 GAAAGCAGTCGCACCGCGTCTATCCATCAGTTATTTATCGCATGACCATTTCATTATTCA

TxVir-CCp5 GAAAGCAGTCGCACCGCGTCTATCCATCAGTTATTTATCGCATGACCATTTCATTATTCA

**** ** ***************** ************************* **** *

Mo7-CCp5 CACCGCTATGAATGGAGTTTTGGCCTTTATGGATGATGACACAGCATTTTTGAACGACTT

L17-CCp5 CACTGCTATGAATGGAGTTTTGGCCTTTATGGATGATGACACAGCATTTTTGAACGACTT

TxAt-CCp5 CACTGCTATGAATGGAGTTTTGGCCTTTATGGATGATGACACAGCATTTTTGAATGATTT

TxVir-CCp5 CACTGCTATGAATGGAGTTTTGGCCTTTATGGATGATGACACAGCATTTTTGAATGATTT

*** ************************************************** ** **

Mo7-CCp5 ATCAAATGACCTTATAGGCACTACATTGTTGCGTACACTTCGTTCACCAGGATACCGGAC

L17-CCp5 ATCAAATGACCTTATAGGCACTACATTGTTGCGTACACTTCGATCACCTGGCTACCGGAC

TxAt-CCp5 ATCAAATGACCTCATAGGCACTACATTGTTGCGTACACTTCGATCACCAGGCTACCGGAC

TxVir-CCp5 ATCAAATGACCTCATAGGCACTACATTGTTGCGTACACTTCGATCACCAGGCTACCGGAC

************ ***************************** ***** ** ********

Mo7-CCp5 GTTTGTGCTACAGGTTAATGTTACTTGTGCTATATACGTTGGATATATATCTGACGTGAT

L17-CCp5 GTTTGTATTACAACTTAATGTTACCTGTGGGATATATGTGGGATATATTTCTGACGTGAT

TxAt-CCp5 GTTTGTATTACAACTTAATGTTACCTGTGCTATATACGTTGGATATATATCTGACGTGAT

TxVir-CCp5 GTTTGTATTACAACTTAATGTTACCTGTGCTATATACGTTGGATATATATCTGACGTGAT

****** **** ********** **** ***** ** ******** ***********

Mo7-CCp5 ACCCATAAGCACTGTGACTGAAGATGCCCTTGAGTTTATTGGATGCCCTGGGTCATTAGT

L17-CCp5 ACCCATAAGCACTGTGACTGAGGACGCCCTCGAGTTTATTGGGTGTCCTGGGTCATTAGT

TxAt-CCp5 ACCCATAAGCACTGTGACTGAAGACGCCCTCGAGTTTATTGGATGTCCTGGATCATTAGT

TxVir-CCp5 ACCCATAAGCACTGTGACTGAAGACGCCCTCGAGTTTATTGGATGTCCTGGATCATTAGT

********************* ** ***** *********** ** ***** ********

Mo7-CCp5 ACACCTCGTTGATGGCACTGATGCCCCTAAACGTCTACATTTAAAGCGTGGTAAACTACA

L17-CCp5 ACATCTCGTTGATGGTACTGATGCCCCCAAACGTCTACATTTAAAGCGTGGTAAACTACA

TxAt-CCp5 ACATCTAGTTGATGGCACGGACGCCCCCAAACGTCTACATTTAAAGCGCGGTAAACTACA

TxVir-CCp5 ACATCTAGTTGATGGCACGGACGCCCCCAAACGTCTACATTTAAAGCGCGGTAAACTACA

*** ** ******** ** ** ***** ******************** ***********

Mo7-CCp5 GCGTGGCCATACTTATACATTCCACGTGGTGGTAAAGAATTCACCGTTCTTTATATTCCT

L17-CCp5 GCGTGGCCATACTTATACATTCCACGTGGAGGTAAAGAATTCACCGTTCTTTATATTCCT

TxAt-CCp5 GCGTGGCCATACTTATACATTCCACGTGGAGGTAAAGAATTCACCGTTCTTTATATTCCT

TxVir-CCp5 GCGTGGCCATACTTATACATTCCACGTGGAGGTAAAGAATTCACCGTTCTTTATATTCCT

***************************** ******************************

Mo7-CCp5 ATCGATGCGTGACTTTGCCGAGCCTTTACAAGGTGAGGGACCAATTAAGGTACTATCTAT

L17-CCp5 ATCGATGCGTGACTTTTCTGAGCCTTTACAGGGTGAGGGACCAATTAAGGTATTATCCAT

TxAt-CCp5 ATCGATGCGTGACTTTTCTGAGCCTTTACAGGGTGAGGGTCCAATTAAGGTATTATCTAT

TxVir-CCp5 ATCGATGCGTGACTTTTCTGAGCCTTTACAGGGTGAGGGTCCAATTAAGGTATTATCTAT

**************** * *********** ******** ************ **** **

Mo7-CCp5 TCCAGACACCGAGTTATACCTCAGCTGTTCTGAGTCCTCTAGTATAGGTGATGGCTTCGG

L17-CCp5 TCCAGACACCGAGTTATACCTCAGTTGTTCTGAGTCCTCTAGTATAGGTGATGGCTTTGG

TxAt-CCp5 TCCTGACACCGAGTTATACCTCAGCTGTTCTGAGTCCTCTAGTATAGGTGATGGCTTCGG

TxVir-CCp5 TCCTGACACCGAGTTATACCTCAGCTGTTCTGAGTCCTCTAGTATAGGTGATGGCTTCGG

*** ******************** ******************************** **

Mo7-CCp5 GTGCACTGCTGGTTTATCTGGTAAACTGCTTGACCAGAAACACGCCATTTGGCGTCCAAC

L17-CCp5 GTGCACTGCTGGTTTATCTGGGAAACTGCTTGACCAAAAACACGCCATTTGGCGTCCAAC

TxAt-CCp5 GTGCACTGCTGGTTTATCTGGTAAGCTACTTGACCAGAAACACGCCATTTGGCGTCCAAC

TxVir-CCp5 GTGCACTGCTGGTTTATCTGGTAAGCTACTTGACCAGAAACACGCCATTTGGCGTCCAAC

********************* ** ** ******** ***********************

Mo7-CCp5 TGAGGGCCCTGGATCATGGTTAAAGTTGGTATTCCGTCGTCCTGTTTTGGTTACTGGATT

L17-CCp5 CGAGGGTCCTGGATCGTGGTTAAAGTTAGTATTCCGTCGTCCTGTACTGGTTACTGGATT

TxAt-CCp5 CGAAGGTCCTGGATCGTGGTTAAAGTTAGTATTCCGTCGTCCTGTACTGGTTACTGGATT

TxVir-CCp5 CGAGGGTCCTGGATCGTGGTTAAAGTTAGTATTCCGTCGTCCTGTACTGGTTACTGGATT

** ** ******** *********** ***************** *************

Mo7-CCp5 CCAGATAAAGCAGAGTGACGACCCTTCTCGATGGGTTCGTCGGATATCACTTGACTACGA

L17-CCp5 CCAGATAAAGCAGAGTGACGACCCTTCTCGATGGGTTCGTCGGATATCACTTGACTACGA

TxAt-CCp5 CCAGATAAAGCAGAGCGACGACCCTTCTCGATGGGTTCGTCGGATATCACTTGACTACGA

TxVir-CCp5 CCAGATAAAGCAGAGCGACGACCCTTCTCGATGGGTTCGTCGGATATCACTTGACTACGA

*************** ********************************************

Mo7-CCp5 TGACGGTACTGAATTTTTCGAGCTTTTGCATTCAAATGACCCTAGGTCTAATTTATACCA

L17-CCp5 TGACGGTACTGAATTTTTCGAGCTTTTGCATTCAAATGACCCTAGGTCTAATTTATATCA

TxAt-CCp5 TGACGGTTCTGAATTTTTCGAGCTTTTGCATTCAAATGACCCTAGGTCTAATTTATACCA

TxVir-CCp5 TGACGGTTCTGAATTTTTCGAGCTTTTGCATTCAAATGACCCTAGGTCTAATTTATACCA

******* ************************************************* **

Mo7-CCp5 CCTAAATGAGCCTCGTTCATTGAATCAGGTTATACTACGTATCGATGAGCTCTACACCAG

L17-CCp5 CCTAAATGAGCCTCGTTCATTGAATCAGGTTATACTACGTATCGATGGGCTCTACACCAG

TxAt-CCp5 CCTAAATGAGCCTCGTTCATTGAATCAGGTCGTACTACGTATCGATGAGCTCTACACCAG

TxVir-CCp5 CCTAAATGAGCCTCGTTCATTGAATCAGGTCATACTACGTATCGATGAGCTCTACACCAG

****************************** *************** ************

Mo7-CCp5 TGCCCAGTCTACTGCCTTGGGTATTAATTTCCTGGGTCAGATGCTATCTGAAGAGTCAGC

L17-CCp5 TGCCCAGTCTACTGCCTTAGGTATTAATTTCCTGGGTCAGATGCTATCTGAGGAGTCAGC

TxAt-CCp5 TGCCCAGTTTACTGCCTTAGGTATTAATTTCCTGGGTCAGATGCTATCTGAGGAGCCAGC

TxVir-CCp5 TGCCCAGTCTACTGCCTTAGGTATTAATTTCCTGGGTCAGATGCTATCTGAGGAGTCAGC

******** ********* ******************************** *** ****

Mo7-CCp5 AGGACTTCGTCGCGTATTAATGAATTGTCATGATACATTATCTGACAATATTGATGTCCA

L17-CCp5 AGGACTTCGTCGCGTGCTAATGAACTGTCATGATACATTATCTGACAACGTTGATGTCCA

TxAt-CCp5 AGGACTTCGTCGCGTGCTAATGAATTGTCATGATACATTATCTGACAACATTGATGTCCA

TxVir-CCp5 AGGACTTCGTCGCGTGCTAATGAATTGTCATGATACATTATCTGACAACATTGATGTCCA

*************** ******* *********************** **********

Mo7-CCp5 GCCTTTATCTGAGGGTCATTCTTACGAACTTGTTTGTAGTCGTCGCTGCTTTGATGTATC

L17-CCp5 GCCTTTATCTGAGGGTCATTCTTACGAGCTTGTTTGCAGTCGTCGCTGCTTTGATGTATC

TxAt-CCp5 GCCTTTATCTGAGGGTCATTCGTACGAGCTTGTCTGTAGTCGTCGCTGCTTTGATGTATC

TxVir-CCp5 GCCTTTATCTGAGGGTCATTCGTACGAGCTTGTCTGTAGTCGTCGCTGCTTTGATGTATC

********************* ***** ***** ** ***********************

Mo7-CCp5 ATCTCCAATGGGTAAAGTCGAGGTTCATGACATTTCTAAACCGCCGTGCGCCGCGCTTAA

L17-CCp5 ATCTCCATTGGGTAAGGTCGAGGTTCATGACATTTCTAAACCGCCGTGTACCGCGCTTAA

TxAt-CCp5 ATCTCCATTGGGCAAAGTCGAGGTACATGACATTTCTAAACCGCCGTGCGCCGCTCTTAA

TxVir-CCp5 ATCTCCATTGGGCAAAGTCGAGGTACATGACATTTCTAAACCGCCGTGCGCCGCTCTTAA

******* **** ** ******** *********************** **** *****

Mo7-CCp5 TATTGACTTATGTCGTGCTGGTGGTGATCACTCAGTATGCCACGCTGCAATAACTATAAT

L17-CCp5 TATTGACTTATGTCGTGCTGGTGGCGATCACTCAGTATGTCACGCTGCAATAACTATAAT

TxAt-CCp5 TATTGACTTATGTCGTGCTGGTGGTGATCACTCAGTATGCCACGCTGCAATAACTATAAT

TxVir-CCp5 TATTGACTTATGTCGTGCTGGTGGTGATCACTCAGTATGCCACGCTGCAATAACTATAAT

************************ ************** ********************

Mo7-CCp5 GAAGAAATCGGAACCCGGTACTGACGATGTATTTGGTTATGTGTTATCAAGGCTTGATCG

L17-CCp5 GAAGAAATCGGAACCCGGTACTGACGATGTATTTGGTTATGTGTTATCAAGGCTTGATCG

TxAt-CCp5 GAAGAAATCTGAACCCGGTACTGACGATGTATTTGGTTATGTGTTATCAAGGCTTGATCG

TxVir-CCp5 GAAGAAATCTGAACCCGGTACTGACGTTGTATTTGGTTATGTGTTATCAAGGCTTGATCG

********* **************** *********************************

Mo7-CCp5 TGGTGTTCGTGATGTACCTTTCCGTGCATCTATTTTGTTCAAGACGGGTACTAACAGATT

L17-CCp5 TGGTGTTCGTGATGTACCTTTCCGTGCATCTATTTTATTTAAGACGGGTACTAACAGATT

TxAt-CCp5 TGGTGTTCGTGATGTACCTTTTCGTGCATCTATTTTGTTTAAAACGGGTACCAACAGATT

TxVir-CCp5 TGGTGTTCGTGATGTACCTTTTCGTGCATCTATTTTGTTTAAAACGGGTACCAACAGATT

********************* ************** ** ** ******** ********

Mo7-CCp5 CCCACAGCATAACTTCTTTGTGGACAACGGCTCTTTGAAGGGTCCTGTGGATGTTTCTTA

L17-CCp5 CCCACAACACAACTTCTTTGTGGACAACGGTTCTTTGAAGGGTCCTGTGGATGTTTCTTA

TxAt-CCp5 CCCACAGCATAACTTCTTTGTGGACAACGGTTCTTTGAAGGGTCCTGTGGATACTTCTTA

TxVir-CCp5 CCCACAGCATAACTTCTTTGTGGACAACGGTTCTTTGAAGGGTCCTGTGGATACTTCTTA

****** ** ******************** ********************* ******

Mo7-CCp5 TATTCGTCAAATTGGTGCCAACCCTCGTATGGGTGAGGTATCATACGGATGGCTTCGTCC

L17-CCp5 TATTCGTCAAATTGGTGCCAACCCTCGTATGGGTGAGGTATCATACGGATGGCTTCGTCC

TxAt-CCp5 TATTCGTCAAATTGGTGCCAACCCTCGTATGGGTGAGGTATCATACGGATGGCTTCGACC

TxVir-CCp5 TATTCGTCAAATTGGTGCCAACCCTCGTATGGGTGAGGTATCATACGGATGGCTTCGACC

********************************************************* **

Mo7-CCp5 TAATGTAAGTGACGTCTACGAGCTGGGTATAGCATTGCCATTACCTGTGGAATCTCAAAA

L17-CCp5 TAATGTAAGTGACACCTACGAGCTGGGCATAGCATTGCCGTTACCTGTGGAATCTCAAAA

TxAt-CCp5 TAATGTAAGTGACGTCTACGAGCTGGGCATAGCATTGCCATTACCTGTGGAATCTCAGAA

TxVir-CCp5 TAATGTAAGTGGCGTCTACGAGCTGGGCATAGCATTGCCATTACCTGTGGAATCTCAGAA

*********** * ************ *********** ***************** **

Mo7-CCp5 ATGCATTCAAACTCCTGGGTGCCAGCCTAACTTCTGGTCTATTGATTTACCTCGCAATGG

L17-CCp5 ATGCATTCAAACTCCTGGATGCCAGCCTAACTTCTGGTCTATTGATTTACCTCGCAATGG

TxAt-CCp5 GTGCATTCAAACTCCTGGGTGCCAGCCTAACTTCTGGTCTATTGATTTACCTCGCAATGG

TxVir-CCp5 GTGCATTCAAACTCCTGGGTGCCAGCCTAACTTCTGGTCTATTGATTTACCTCGCAATGG

***************** *****************************************

Mo7-CCp5 CCGCTATAAGATCGAGTTAGTTTTGGGCAATTCGGTATCTTCGGATGTAACTACTGTGGA

L17-CCp5 TCGATATAAGATCGAGTTAGTTTTGGGCAATTCGGTATCTTCGGATGTAACTACTGTGGA

TxAt-CCp5 CCGCTACAAGATCGAGTTAGTTTTGGGCAATTCGGTATCTTCGGATGTAACTACTGTGGA

TxVir-CCp5 CCGCTACAAGATCGAGTTAGTTTTGGGCAATTCGGTATCTTCGGATGTAACTACTGTGGA

** ** *****************************************************

Mo7-CCp5 TGACATAGTTCCCAATGGCGCCTTGGTTCCGCAGCGTCTGTGTTTGGAGGTTAATGGTCA

L17-CCp5 TGACATAGTTCCCAATGGCGCCTTGGTTCCGCAGCGTCTGTGTTTGGAGGTTAATGGTCA

TxAt-CCp5 CGACATAGTTCCCAATGGCGCTTTGGTTCCTCAGCGTCTGTGTTTGGAGGTTAATGGTCA

TxVir-CCp5 CGACATAGTTCCCAATGGCGCTTTGGTTCCTCAGCGTCTGTGTTTGGAGGTTAATGGTCA

******************** ******** *****************************

Mo7-CCp5 GGTAGTCCTCAACTGTGTTGAGATTGCTGGCGGTTCACTTTACACCTTGGTCAAGGAGAT

L17-CCp5 GGTAGTCCTCAACTGTGTTGAGATTGCTGGCGGTTCACTTTACACCTTGGTCAAGGAGAT

TxAt-CCp5 GGTAGTCCTCAACTGTGTTGAGATTGCTGGCGGTTCACTTTACACCTTGGTCAAGGAGAT

TxVir-CCp5 GGTAGTCCTCAACTGTGTTGAGATTGCTGGCGGTTCACTTTACACCTTGGTCAAGGAGAT

************************************************************

Mo7-CCp5 TGAGGTGCGCAACAACTTGCTGAAGTTGACTTCTACTAGCAGCAACAGCTCG

L17-CCp5 TGAGGTGCGCAACAACTTGCTGAAGTTGACTTCTACTAGCAGCAACAGCTCG

TxAt-CCp5 TGAGGTGCGCAACAACTTGCTGAAGTTGACTTCTACTAGCAGCAACAGCTCG

TxVir-CCp5 TGAGGTGCGCAACAACTTGCTGAAGTTGACTTCTACTAGCAGCAACAGCTCG

****************************************************

Mo7-FNPA AAACCCTTTTGTATTACTCATTGCTAAGTGTGAGATTCTTATACTGCGTGTTCGTGTTTC

TxVir2-FNPA AAACCCTTTCGTATTATTCATTGCTAAGTGTGAGATTCTTATACTGCGTTTTCGTGTTTC

L17-FNPA AAACCCTTTCGTATTATTCATTGCTAAGTGTGAGATTCTTATACTGCGTTTTCGTGTTTC

TxAt-FNPA AAACCCTTTCGTATTATTCATTGCTAAGTGTGAGATTCTTATACTGCGTTTTCGTGTTTC

********* ****** ******************************** **********

Mo7-FNPA ATACTGTATTTGCCAATGGAGATATAAATAAGGATGTTGTTTCACCTAAGGATTTCACCA

TxVir2-FNPA ATACTGTATTTGCCAATGGAGATATAAATCAGGATGTTGTTTCACCTAAGGATTTCACCA

L17-FNPA ATACTGTATTTGCCAATGGAGATATAAATCAGGATGTTGTTTCACCTAAGGATTTCACCA

TxAt-FNPA ATACTGTATTTGCCAATGGAGATATAAATCAGGATGTTGTTTCACCTAAGGATTTCACCA

***************************** ******************************

Mo7-FNPA TACCTTCAGGAACCGACTATGATCGGGAAGCCTTAATGGTAATTTGTATATACTTCATAA

TxVir2-FNPA TACCTTCAGGAACCGACTATGATCGGGAAGCCTTAATGGTAATTTGTATATACTTCACAA

L17-FNPA TACCTTCAGGAACCGACTATGATCGGGAAGCCTTAATGGTAATTTGTATATACTTCACAA

TxAt-FNPA TACCTTCAGGAACCGACTATGATCGGGAAGCCTTAATGGTAATTTGTATATACTTCACAA

********************************************************* **

Mo7-FNPA CACTTATTTTACGTAGAAATTGACCGAATTTAGACAACGTCACAGGAAGACCGTTGATGG

TxVir2-FNPA TACTTATTTTACGTAGAAATTGACCGAATTTAGACAACGTCACAGGAAGACCGTTGATGG

L17-FNPA TACTTATTTTACGTAGAAATTGACCGAATTTAGACAACGTCACAGGAAGACCGTTGATGG

TxAt-FNPA TACTTATTTTACGTAGAAATTGACCGAATTTAGACAACGTCACAGGAAGACCGTTGATGG

***********************************************************

Mo7-FNPA TATGCTTTGTGCAGCAGCTTTTGTGCGCAATGGGAATACATTTACGGACTGTACAACAAC

TxVir2-FNPA TATGCTTTGTGCAGCAGCTTTTGTGCGCAATGGGAATACATTTACGGACTGTACAACAAC

L17-FNPA TATGCTTTGTGCAGCAGCTTTTGTGCGCAATGGGAATACATTTACGGACTGTACAACAAC

TxAt-FNPA TATGCTTTGTGCAGCAGCTTTTGTGCGCAATGGGAATACATTTACGGACTGTACAACAAC

************************************************************

Mo7-FNPA TGAAGCTCCAGATGGTTCTGTCGGTAGAGAATGGTGTTATGTAGAAGTACAGTTGATTGG

TxVir2-FNPA TGAAGCTCCAGATGGTTCTGTTGGTCGAGAATGGTGTTATGTAGAAGTACAGTTGATTGG

L17-FNPA TGAAGCTCCAGATGGTTCTGTTGGTCGAGAATGGTGTTATGTAGAAGTACAGTTGATTGG

TxAt-FNPA TGAAGCTCCAGATGGTTCTGTTGGTCGAGAATGGTGTTATGTAGAAGTACAGTTGATTGG

********************* *** **********************************

Mo7-FNPA TGTGGGCTCCAGGGATTGGGACTTTTGTGCCGGCACAGTAGACTATGATGTCATTCGATC

TxVir2-FNPA TGTGGGCTCCAGAGATTGGGACTTTTGTGCCGGCACAGTAGACTATGATGTCATTCGATC

L17-FNPA T-TGGGCTCCAGAGATTGGGACTTTTGTGCCGGCACAGTAGACTATGATGTCATTCGATC

TxAt-FNPA TGTGGGCTCCAGAGATTGGGACTTTTGTGCCGGCACAGTAGACTATGATGTCATTCGATC

* ********** ***********************************************

Mo7-FNPA CAAGGCATCACTTTTATCGAAACTGAAATCCGATGAGTTGGGACATTCCGTGTTACAACT

TxVir2-FNPA CAAGGCATCTCTTTTATCGAAACTGAAATCTGAAGAGTTGGCACATTCAGTGTTACAACT

L17-FNPA CAAGGCATCTCTTTTATCGAAACTGAAATCTGAAGAGTTGGCACATTCAGTGTTACAACT

TxAt-FNPA CAAGGCATCTCTTTTATCGAAACTGAAATCTGAAGAGTTGGCACATTCAGTGTTACAACT

********* ******************** ** ******* ****** ***********

Mo7-FNPA TTCTGCAACTGAACAGTAGGTTATAATCTTTTTGTTAAAGATCCACAGACGTTTGGAAAA

TxVir2-FNPA TTCTGCAACTGAACAGTAGGTTATAATCTTTTTGTTAAAGATCCGCAGACGTTTGGAAAA

L17-FNPA TTCTGCAACTGAACAGTAGGTTATAATCTTTTTGTTAAAGATCCGCAGACGTTTGGAAAA

TxAt-FNPA TTCTGCAACTGAACAGTAGGTTATAATCTTTTTGTTAAAGATCCGCAGACGTTTGGAAAA

******************************************** ***************

Mo7-FNPA TACGCTCAAACATTTTGATGCGGTCTGTGGTCACGGAACGGCATCTCACGAAAATGATAT

TxVir2-FNPA TACGCTAAAACATTTTGATGCTGTGTGTGGCCACGGAACGGCATCTCATGAAAATGATAT

L17-FNPA TACGCTAAAACATTTTGATGCTGTGTGTGGCCACGGAACGGCATCTCATGAAAATGATAT

TxAt-FNPA TACGCTAAAACATTTTGATGCTGTGTGTGGCCACGGAACGGCATCTCATGAAAATGATAT

****** ************** ** ***** ***************** ***********

Mo7-FNPA GAACACAATATCGCATTCTCTCCGTGGTTTGGAGCGTGCTTTACAGCAGGTTGAGGCAAA

TxVir2-FNPA GAACACAATATCGCACTCTCTCCGTGGTTTGGAGCGTGCTTTACAGCAGGTTGAGGCAAA

L17-FNPA GAACACAATATCGCACTCTCTCCGTGGTTTGGAGCGTGCTTTACAGCAGGTTGAGGCAAA

TxAt-FNPA GAACACAATATCGCACTCTCTCCGTGGTTTGGAGCGTGCTTTACAGCAGGTTGAGGCAAA

*************** ********************************************

Mo7-FNPA CTCACAAAGTCTCCACATGTTAAGTGAGGAATACGAATCTCTGAGCGAGCAGCTTGAGAT

TxVir2-FNPA CTCACAAAGTCTCCACATGTTAAATGAGGAATACGAATCTCTGAGCGAGCAGCTTGAGAT

L17-FNPA CTCACAAAGTCTCCACATGTTAAATGAGGAATACGAATCTCTGAGCGAGCAGCTTGAGAT

TxAt-FNPA CTCACAAAGTCTCCACATGTTAAATGAGGAATACGAATCTCTGAGCGAGCAGCTTGAGAT

*********************** ************************************

Mo7-FNPA TACGCGTAAGAGCGTTTTAAATGACAAAAGAAATTGCTCAATTGTGCAAGGCTATTCCGC

TxVir2-FNPA TACGCGTAAGAGCGTTTTAAATGACAAAAGAAATTGCTCAATTGTTCAAGGCTATTCCGT

L17-FNPA TACGCGTAAGAGCGTTTTAAATGACAAAAGAAATTGCTCAATTGTTCAAGGCTATTCCGT

TxAt-FNPA TACGCGTAAGAGCGTTTTAAATGACAAAAGAAATTGCTCAATTGTTCAAGGCTATTCCGT

********************************************* *************

Mo7-FNPA TGCGGTAGGTGATACATGGGTGGTAAATTCATTATTGGTATATAGGTGGTGGGAGATGGC

TxVir2-FNPA TGCGGTAGGTGATACATGGGTGGTAAATACATTATTGTTATATAGGTGGNGGGAGATGGT

L17-FNPA TGCGGTAGGTGATACATGGGTGGTAAATACATTATTGTTATATAGGTGGTGGGAGATGGT

TxAt-FNPA TGCGGTAGGTGATACATGGGTGGTAAATACATTATTGTTATATAGGTGGTGGGAGATGGT

**************************** ******** *********** *********

Mo7-FNPA GTCCGTGCCAGCTACTTTGACAATCCATATCTAAGGGGACCACCTGTTGGTTTCTTTGAT

TxVir2-FNPA GTCCGTGCCAGCTACTTTGACAATCCATATCTAAGGGGGCCACCTGTTGGTTTCTTTGAT

L17-FNPA GTCCGTGCCAGCTACTTTGACAATCCATATCTAAGGGGGCCACCTGTTGGTTTCTTTGAT

TxAt-FNPA GTCCGTGCCAGCTACTTTGACAATCCATATCTAAGGGGGCCACCTGTTGGTTTCTTTGAT

************************************** *********************

Mo7-FNPA CATTCTAGCCTATCTCTAACATTTGATGAGATATTACCAATAAATGGTGTAGACATTCGT

TxVir2-FNPA CATTCTAGCCTATCTCTAACATTTGATGAGATATTACCTATAAATGGTGTAGACATTCGT

L17-FNPA CATTCTAGCCTATCTCTAACATTTGATGAGATATTACCTATAAATGGTGTAGACATTCGT

TxAt-FNPA CATTCTAGCCTATCTCTAACATTTGATGAGATATTACCTATAAATGGTGTAGACATTCGT

************************************** *********************

Mo7-FNPA TCCTTTTCCGTACGTTTTGAGACATATTTGCGCGTACCTGTTTCGGGAGACTATACCTTT

TxVir2-FNPA TCCTTTTCCGTACGTTTTGAGACATATTTGCGCGTACCTGTTTCGGGAGACTATACCTTT

L17-FNPA TCCTTTTCCGTACGTTTTGAGACATATTTGCGCGTACCTGTTTCGGGAGACTATACCTTT

TxAt-FNPA TCCTTTTCCGTACGTTTTGAGACATATTTGCGCGTACCTGTTTCGGGAGACTATACCTTT

************************************************************

Mo7-FNPA TGGTTGGATGCTGATTGTAACTTTTTGATGTTCGTAGACGGAGAGTTGGTAATTAAGCAT

TxVir2-FNPA TGGTTGGATGCTGATTGTAACTTTTTGATGTTCGTCGACGGAGAGTTGGTAATTAAGCAT

L17-FNPA TGGTTGGATGCTGATTGTAACTTTTTGATGTTCGTCGACGGAGAGTTGGTAATTAAGCAT

TxAt-FNPA TGGTTGGATGCTGATTGTAACTTTTTGATGTTCGTCGACGGAGAGTTGGTAATTAAGCAT

*********************************** ************************

Mo7-FNPA GGACTTGAGGGTAGGGGTACATTTGCGTCGGCGTATAATTCAACGGGTGTGGTTGCTCTT

TxVir2-FNPA GGACTTGAGGGTAGGGGTACATTTGCATCGGCGTATAATTCAGCGGGTGTGGTTGCTCTT

L17-FNPA GGACTTGAGGGTAGGGGTACATTTGCATCGGCGTATAATTCAGCGGGTGTGGTTGCTCTT

TxAt-FNPA GGACTTGAGGGTAGGGGTACATTTGCATCGGCGTATAATTCAGCGGGTGTGGTTGCTCTT

************************** *************** *****************

Mo7-FNPA GACGGCCGTACAACAACCTCGACTCTCGTTTCTTCTCGCAAAATGTCACTGGTTGGGGGC

TxVir2-FNPA GACGGCCGTACAACAACCTCGACTCTCGTTTCTTCTCGCAAAATGTCACTGGTTGGGGGC

L17-FNPA GACGGCCGTACAACAACCTCGACTCTCGTTTCTTCTCGCAAAATGTCACTGGTTGGGGGC

TxAt-FNPA GACGGCCGTACAACAACCTCGACTCTCGTTTCTTCTCGCAAAATGTCACTGGTTGGGGGC

************************************************************

Mo7-FNPA AAGCGTTATCCATTGGTTTTGGAGTATTCACATCAAAGCTCACTGAAGTATCGTGATGAA

TxVir2-FNPA AAGCGTTATCCATTGGTATTGGAATATTCACATCAAAGCTCACTGAAGTATCGTGATGAA

L17-FNPA AAGCGTTATCCATTGGTATTGGAATATTCACATCAAAGCTCACTGAAGTATCGTGATGAA

TxAt-FNPA AAGCGTTATCCATTGGTATTGGAATATTCACATCAAAGCTCACTGAAGTATCGTGATGAA

***************** ***** ************************************

Mo7-FNPA AACGTTGGTCGTTTAGTATTGAGTTGGGAAACTGACATGCGGTCCCGGACGATTATCGAC

TxVir2-FNPA AACGTTGTTCGTTTGATATTGAGTTGGGAAACTGACACGCGGTCCCGGACGATTATCGAC

L17-FNPA AACGTTGTTCGTTTGATATTGAGTTGGGAAACTGACACGCGGTCCCGGACGATTATCGAC

TxAt-FNPA AACGTTGTTCGTTTGATATTGAGTTGGGAAACTGACACGCGGTCCCGGACGATTATCGAC

******* ****** ********************* **********************

Mo7-FNPA CCGGACTATTTCTTCCGTAGTAGAGATTCTGGTGAATCTTTAATTATCAGTGGATTAGAT

TxVir2-FNPA CCGGATTATTTCTTCCGTAGCAGAGATTCTGGTGAATCTTTAATTATCAGTGGATTAGAT

L17-FNPA CCGGATTATTTCTTCCGTAGCAGAGATTCTGGTGAATCTTTAATTATCAGTGGATTAGAT

TxAt-FNPA CCGGATTATTTCTTCCGTAGCAGAGATTCTGGTGAATCTTTAATTATCAGTGGATTAGAT

***** ************** ***************************************

Mo7-FNPA GCGCGATGGTTTGACTTAGCCATGTTGGAGAACGGGGCACAGGCATTCATTAACGTGACC

TxVir2-FNPA GCGCGATGGTTTGACTTAGCCATGTTGGAGAACGGGGCACAGGCATTTATTAACGTAACA

L17-FNPA GCGCGATGGTTTGACTTAGCCATGTTGGAGAACGGGGCACAGGCATTTATTAACGTAACA

TxAt-FNPA GCGCGATGGTTTGACTTAGCCATGTTGGAGAACGGGGCACAGGCATTTATTAACGTAACA

*********************************************** ******** **

Mo7-FNPA AATTTGTTGTTGGCAGACGTACCTGACCATCTCCGAGGCATGCGTATGGTACGCACAGTA

TxVir2-FNPA AATTTGTTGTTGGCAGACGTACCTGACCATCTCCGAGGCATGCGTATGGTACGCACAGTA

L17-FNPA AATTTGTTGTTGGCAGACGTACCTGACCATCTCCGAGGCATGCGTATGGTACGCACAGTA

TxAt-FNPA AATTTGTTGTTGGCAGACGTACCTGACCATCTCCGAGGCATGCGTATGGTACGCACAGTA

************************************************************

Mo7-FNPA GTGAAACCTGATTATGATACCGTGGATTTTTACATTTCCCATGATGCTTTTCTATATGTT

TxVir2-FNPA GTGAAACCTGATTATGATACCGTGGATTTTTACATTTCGCATGATGCTTTTCTATATGTT

L17-FNPA GTGAAACCTGATTATGATACCGTGGATTTTTACATTTCGCATGATGCTTTTCTATATGTT

TxAt-FNPA GTGAAACCTGATTATGATACCGTGGATTTTTACATTTCGCATGATGCTTTTCTATATGTT

************************************** *********************

Mo7-FNPA GCTCAATCTGAACATCTGTCTTATATTCCTATGGCAGAGAATATGTCTATGTTCGATCGA

TxVir2-FNPA GCTCAATCTGAACATCTGTCTTATATTCCTATGGCAGAGAATATGTCCACGTTCGATAGA

L17-FNPA GCTCAATCTGAACATCTGTCTTATATTCCTATGGCAGAGAATATGTCCACGTTCGATAGA

TxAt-FNPA GCTCAATCTGAACATCTGTCTTATATTCCTATGGCAGAGAATATGTCCACGTTCGATAGA

*********************************************** * ******* **

Mo7-FNPA AGCTGGGAGGTGATATCAGTATATTCTATTGGCCACAATTGCGAAGAGGCCATCAGTCAA

TxVir2-FNPA AGCTGGGAGGTGATATCAGTTTATTCTATTGGCCACAATTGCGAAGAGGCCACCAGTCAA

L17-FNPA AGCTGGGAGGTGATATCAGTTTATTCTATTGGCCACAATTGCGAAGAGGCCACCAGTCAA

TxAt-FNPA AGCTGGGAGGTGATATCAGTTTATTCTATTGGCCACAATTGCGAAGAGGCCACCAGTCAA

******************** ******************************* *******

Mo7-FNPA CAGGAGTTTATAATTTTCTACAAGCGATTTAAGGCAGGACCTGTAAAAATCCACATATTA

TxVir2-FNPA CAGGAGTTTATAGTTTTCTACAAGCGATTCAAGGCAGGACCTGTAAAAATTCACATATTA

L17-FNPA CAGGAGTTTATAGTTTTCTACAAGCGATTCAAGGCAGGACCTGTAAAAATTCACATATTA

TxAt-FNPA CAGGAGTTTATAGTTTTCTACAAGCGATTCAAGGCAGGACCTGTAAAAATTCACATATTA

************ **************** ******************** *********

Mo7-FNPA CCCGAAACGTCGTTTTTCTTGTTCATGCAGCCCGCTGCAGCTAATGCAATATGTCCCGAC

TxVir2-FNPA CCTGAAACGTCGTTTTTCTTGTTCATGCAGCCCGCTGCGGCTAATGCCATATGCCCCGAC

L17-FNPA CCTGAAACGTCGTTTTTCTTGTTCATGCAGCCCGCTGCGGCTAATGCCATATGCCCCGAC

TxAt-FNPA CCTGAAACGTCGTTTTTCTTGTTCATGCAGCCCGCTGCGGCTAATGCCATATGCCCCGAC

** *********************************** ******** ***** ******

Mo7-FNPA GATGTCCAGTATCTTCCTTTCAAAAACGGTGACGGTTGTGCGTCTTCTTCATCACTTTCA

TxVir2-FNPA GATGTCCAGTATCTTCCTTTCAAAAATGGTGACGGTTGTGCGTCTTCTTCATCACTTTCA

L17-FNPA GATGTCCAGTATCTTCCTTTCAAAAATGGTGACGGTTGTGCGTCTTCTTCATCACTTTCA

TxAt-FNPA GATGTCCAGTATCTTCCTTTCAAAAATGGTGACGGTTGTGCGTCTTCTTCATCACTTTCA

************************** *********************************

Mo7-FNPA GCCGCATTTGACTGCTCAAAAGCATTTGGGAGTGGTTACTGGCAACCGAGTTCTGGGCGG

TxVir2-FNPA GCTGCATTTGACTGCTCAAAAGCTTTTGGGAGTGGATACTGGCAACCGAGTTCTGGGCGG

L17-FNPA GCTGCATTTGACTGCTCAAAAGCTTTTGGGAGTGGATACTGGCAACCGAGTTCTGGGCGG

TxAt-FNPA GCTGCATTTGACTGCTCAAAAGCTTTTGGGAGTGGATACTGGCAACCGAGTTCTGGGCGG

** ******************** *********** ************************

Mo7-FNPA ATCACTGGACAGTGGCTGATGCGCACTTTCACCAACCCTGTGGAGTTGGTTCACTTCCAT

TxVir2-FNPA ATCACTGGACAGTGGCTGATGCGCACTTTCACCAACCCTGTGGAGTTGGTTCACTTCCAT

L17-FNPA ATCACTGGACAGTGGCTGATGCGCACTTTCACCAACCCTGTGGAGTTGGTTCACTTCCAT

TxAt-FNPA ATCACTGGACAGTGGCTGATGCGCACTTTCACCAACCCTGTGGAGTTGGTTCACTTCCAT

************************************************************

Mo7-FNPA TTTTCTCCGATATATGGTGGTTTACCTATGCACGCAATTATATCATTTCCCGATGGATCT

TxVir2-FNPA TTTTCTCCGATAGATGGCAGTTTACCTATGCGCGCAATTATATCATTTCCTGATGGATCT

L17-FNPA TTTTCTCCGATAGATGGCAGTTTACCTATGCGCGCAATTATATCATTTCCTGATGGATCT

TxAt-FNPA TTTTCTCCGATAGATGGCAGTTTACCTATGCGCGCAATTATATCATTTCCTGATGGATCT

************ **** ************ ****************** *********

Mo7-FNPA GAGGAAGATTTCGAGCTGCATTCACAGCTTCGTTACGAATTGGGATACCACGGCGTTGTG

TxVir2-FNPA GAGGAAGATTTCGAGCTGCATTCACAGCTTCGTTACGAATTGGGATATCACGGCGTTGTG

L17-FNPA GAGGAAGATTTCGAGCTGCATTCACAGCTTCGTTACGAATTGGGATATCACGGCGTTGTG

TxAt-FNPA GAGGAAGATTTCGAGCTGCATTCACAGCTTCGTTACGAATTGGGATATCACGGCGTTGTG

*********************************************** ************

Mo7-FNPA GATTCCATTAGGATCATGATTGAAACAATGAGCCCAGGAGATGGCATTACAGGTTCCGAT

TxVir2-FNPA GATTCCATTAGGATTATGATTGAAACAATGAGCCCAGGAGACGGTATTACAGGTTCCGAT

L17-FNPA GATTCCATTAGGATTATGATTGAAACAATGAGCCCAGGAGACGGTATTACAGGTTCCGAT

TxAt-FNPA GATTCCATTAGGATTATGATTGAAACAATGAGCCCAGGAGACGGTATTACAGGTTCCGAT

************** ************************** ** ***************

Mo7-FNPA ACGGTTGATAATAAAGAAATAATTGGCGGAACATTCGCCTTTTATGGACGCGAATGCGGC

TxVir2-FNPA ACGGTTGATAATAAAGAAATAATTGGCGGAACATTCGCCTTTTATGGACGCGAATGCGGT

L17-FNPA ACGGTTGATAATAAAGAAATAATTGGCGGAACATTCGCCTTTTATGGACGCGAATGCGGT

TxAt-FNPA ACGGTTGATAATAAAGAAATAATTGGCGGAACATTCGCCTTTTATGGACGCGAATGCGGT

***********************************************************

Mo7-FNPA GCTCGTACAACAGTGGAAGAAGCAGTACATTTTCCCATTCACATAAGTTTCTGCCAAGGG

TxVir2-FNPA GCTCGTACAACAGTGGAAGAAGCAGTACATTTTCCCATTCACATAAGTTTCTGCCAAGGG

L17-FNPA GCTCGTACAACAGTGGAAGAAGCAGTACATTTTCCCATTCACATAAGTTTCTGCCAAGGG

TxAt-FNPA GCTCGTACAACAGTGGAAGAAGCAGTACATTTTCCCATTCACATAAGTTTCTGCCAAGGG

************************************************************

Mo7-FNPA GGACACGCTTGTGGTCCTGATCACTTGGATTTGGGACACACGAAAGGATATCATGGCAGG

TxVir2-FNPA GGGCACGCATGTGGTCCTGATCACTTGGATTTGGGACACATGAAAGGATATCATGGCAGG

L17-FNPA GGGCACGCATGTGGTCCTGATCACTTGGATTTGGGACACATGAAAGGATATCATGGCAGG

TxAt-FNPA GGGCACGCATGTGGTCCTGATCACTTGGATTTGGGACACATGAAAGGATATCATGGCAGG

** ***** ******************************* *******************

Mo7-FNPA TTGTCTTACGGTTGGGCTTCATCAAATGTCGTTGCTGATATTGCTGATATTCAGATATGT

TxVir2-FNPA TTGTCTTACGGTTGGGGTTCATCAAATGTCGTGGCTGATATAGCTGATCTTCAGATATGT

L17-FNPA TTGTCTTACGGTTGGGGTTCATCAAATGTCGTGGCTGATATAGCTGATCTTCAGATATGT

TxAt-FNPA TTGTCTTACGGTTGGGGTTCATCAAATGTCGTGGCTGATATAGCTGATCTTCAGATATGT

**************** *************** ******** ****** ***********

Mo7-FNPA AGACCTCAAGTGAATCACATAGTTGATGTTAACTTGGATTCTGTACCGTTGATAGATAGA

TxVir2-FNPA AAACCCCAAGTGAATCACATAGTTGATGTTAACTTGGATTCTGTACCGTTGATAGATAGA

L17-FNPA AAACCCCAAGTGAATCACATAGTTGATGTTAACTTGGATTCTGTACCGTTGATAGATAGA

TxAt-FNPA AAACCCCAAGTGAATCACATAGTTGATGTTAACTTGGATTCTGTACCGTTGATAGATAGA

* *** ******************************************************

Mo7-FNPA GAATTAACAGAACTATCTGCAACGACTAAAAAGCATAGTGTATATGAATTGTTATTGAAG

TxVir2-FNPA GAATTAACAGAACTACCTGCGACGACTAAAAATCATAGTGTATATGATTTGTTATTGAAG

L17-FNPA GAATTAACAGAACTACCTGCGACGACTAAAAATCATAGTGTATATGATTTGTTATTGAAG

TxAt-FNPA GAATTAACAGAACTACCTGCGACGACTAAAAATCATAGTGTATATGATTTGTTATTGAAG

*************** **** *********** ************** ************

Mo7-FNPA CAGAAAGCTGGACTACCTCTTGGCCAGGGGCAACGGTGGACCATCGACGTCCCTGAGCAT

TxVir2-FNPA CAGAAAGCTGGATTACCTCTTGGCCAGGGGCAACGGTGGACCATCGATGTCCCTGAGCAT

L17-FNPA CAGAAAGCTGGATTACCTCTTGGCCAGGGGCAACGGTGGACCATCTATGTCCCTGAGCAT

TxAt-FNPA CAGAAAGCTGGATTACCTCTTGGCCAGGGGCAACGGTGGACCATCGATGTCCCTGAGCAT

************ ******************************** * ************

Mo7-FNPA GGTGTATACAAAGTTGAAGTTTTACTTTCTGCGTTATGTACTAATGTGGATTCTGCGTCT

TxVir2-FNPA GGTGTATACAATGTTGAAGTTTTACTTTCTGCGTTATGTACTAATGTGGAATCTGCGTCC

L17-FNPA GGTGTATACAATGTTGAAGTTTTACTTTCTGCGTTATGTACTAATGTGGAATCTGCGTCC

TxAt-FNPA GGTGTATACAATGTTGAAGTTTTACTTTCTGCGTTATGTACTAATGTGGAATCTGCGTCC

*********** ************************************** ********

Mo7-FNPA CTTCTCATAAA

TxVir2-FNPA CTTCTCATAAA

L17-FNPA CTTCTCATAAA

TxAt-FNPA CTTCTCATAAA

***********

**Supplementary Figure 5**

Percentage of identity of nucleotide (a) and amino acid (b) of *CCp5* and *FNPA* genes among different piroplasm species.


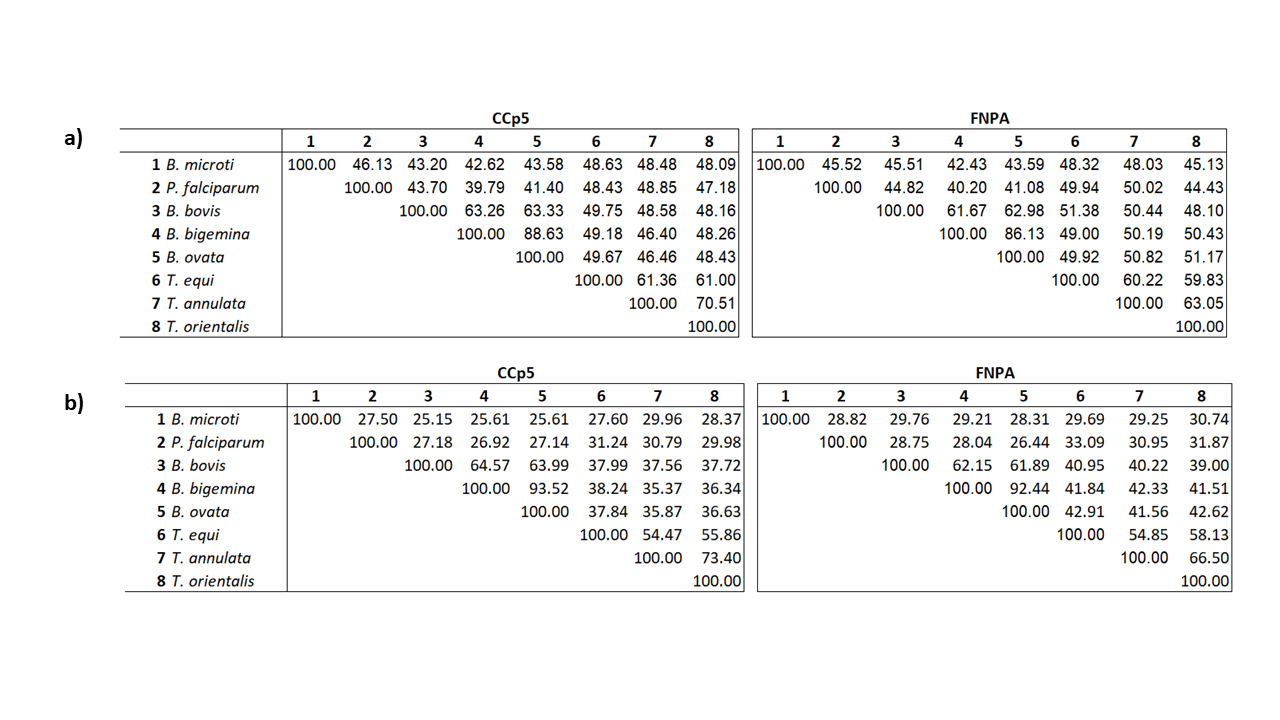


**Supplementary Figure 6:** Control immunofluorescence assays using pre-immune CCp5 (a) and FNPA (b) rabbit sera, performed on blood stage and *in vitro* induced sexual stages (12h, 24h, and 48h) of *B. bovis*.


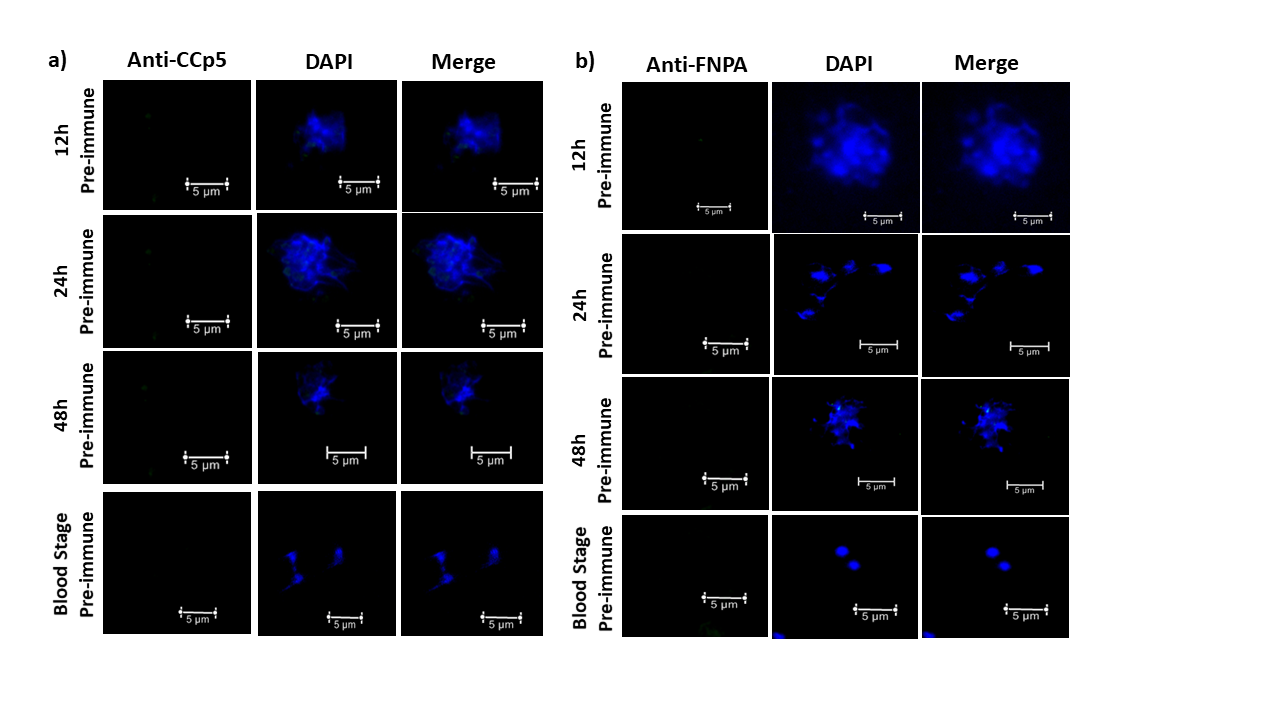

Supplement: Supplementary file 1 [file Data_Sheet_1.docx]
